# Rejuvenating the [1, 2, 3]-triazolo [1,5-a]quinoxalin-4(5*H*)-one scaffold: Synthesis and derivatization in a sustainable guise and preliminary antimicrobial evaluation

**DOI:** 10.3389/fchem.2023.1126427

**Published:** 2023-03-14

**Authors:** Sveva Pelliccia, Antonella Ilenia Alfano, Beatriz Ramos Gomes Da Assunção, Luigia Turco, Francesca Lembo, Vincenzo Summa, Elisabetta Buommino, Margherita Brindisi

**Affiliations:** ^1^ Department of Pharmacy, University of Naples Federico II, Naples, Italy; ^2^ ESTeSL- Lisbon School of Health Technology, Polytechnic Institute of Lisbon, Lisbon, Portugal; ^3^ Department of Precision Medicine, University of Campania “Luigi Vanvitelli”, Naples, Italy

**Keywords:** [1,2,3]-triazolo [1,5-a] quinoxalin-4(5H)-one, privileged scaffold, sustainable synthesis, green chemistry, drug discovery, antimicrobial agents

## Abstract

The [1,2,3]-triazolo [1,5-a] quinoxalin-4(5*H*)-one scaffold and its analogues triazole-fused heterocyclic compounds are relevant structural templates in both natural and synthetic biologically active compounds. However, their medicinal chemistry applications are often limited due to the lack of synthetic protocols combining straightforward generation of the central core while also allowing extensive decoration activity for drug discovery purposes. Herein, we report a “refreshed” synthesis of the [1,2,3]-triazolo [1,5-*a*]quinoxalin-4(5*H*)-one core, encompassing the use of eco-compatible catalysts and reaction conditions. We have also performed a sustainable and extensive derivatization campaign at both the endocyclic amide nitrogen and the ester functionality, comprehensively exploring the reaction scope and overcoming some of the previously reported difficulties in introducing functional groups on this structural template. Finally, we unveiled a preliminary biological investigation for the newly generated chemical entities. Our assessment of the compounds on different bacterial species (two *S. aureus* strains, three *P. aeruginosa* strains, *K. pneumonia*), and two fungal *C. albicans* strains, as well as the evaluation of their activity on *S. epidermidis* biofilm formation, foster further optimization for the retrieved hit compounds **9**, **14**, and **20**.

## 1 Introduction

The triazoloquinoxaline scaffold is considered a versatile moiety, and an important structural template for the design and synthesis of novel biologically relevant compounds such as antibacterial, anti-HIV, antitrypanosomal, antiallergic, antifungal, cardiovascular, antileishmanial, and chemotherapeutic agents (Amer et al., 2010; El-Sagheer and Brown, 2012; Ayoup et al., 2016; Nagavelli et al., 2016; Zhang et al., 2017; Ayoup et al., 2022). Despite several applications of this scaffold in medicinal chemistry and its special features and potentiality of derivatization, little has been done with respect to the search of versatile and potentially ecofriendly synthetic protocols (Baashen et al., 2016).

Accordingly, compared to the more largely explored and synthetically accessible 1,2,4-triazoloquinoxalines (Alswah et al., 2018; El-Attar et al., 2018; Houa et al., 2020; Wesseler et al., 2022), only few applications on the 1,2,3-triazoloquinoxaline counterpart have been reported in literature, most probably due to the available somewhat outdated synthetic protocols that lack versatility for quick scaffold decoration and derivatization.

**SCHEME 1:**
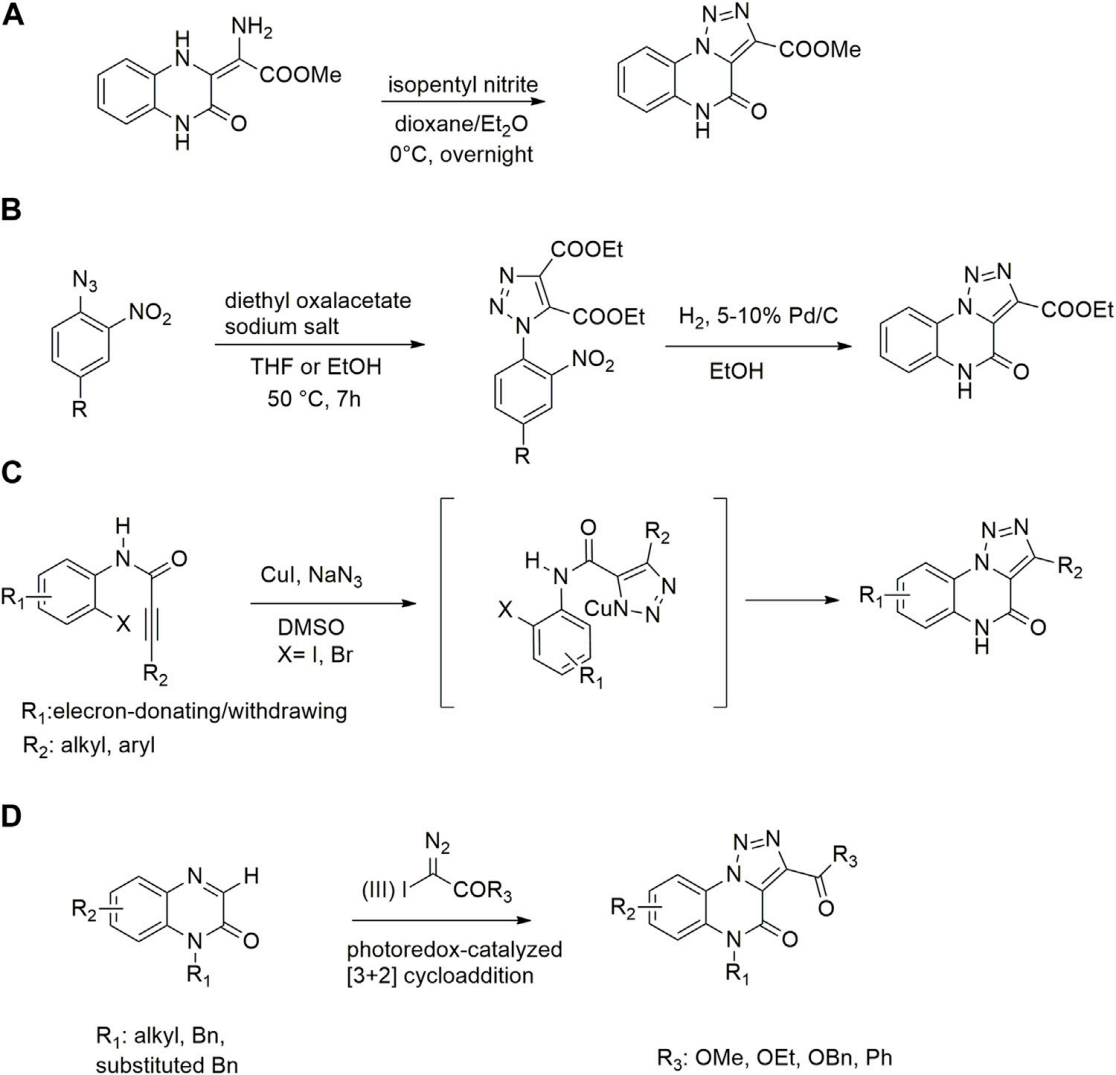
Previously reported approaches for [1,2,3]-triazolo [1,5-*a*] quinoxalin-4(5*H*)-ones synthesis **(A–D)**.

Typically [1,2,3]-triazolo [1,5-*a*] quinoxalin-4(5*H*)-ones were prepared through cyclization of methyl 2-amino-2-(3,4-dihydro)-2-(3,4-dihydro-2(1*H*)-quinoxalinylidene) acetate with amyl nitrite in presence of 2,2,2- trichloroacetic acid and dioxane/diethyl ether as solvents (Scheme 1A) (Ager et al., 1988).

More recent methodologies for the generation of this tricyclic system require multiple steps involving the preparation of the suitable triazole intermediates followed by cycloamidation. Accordingly, substituted 2-nitrophenyl azides can react with diethyl oxalacetate, ethyl benzoyl pyruvate and ethyl 2-furoyl pyruvate in form of their sodium salts producing triazoles diesters in a complex mixture of triazole byproducts, corresponding aniline of the azide and substituted benzofurazan-*N*-oxide (Scheme 1B) (Biagi et al., 2002). 2-Nitrophenyl triazoles subsequently undergo catalytic hydrogenation with Pd/C thus providing the corresponding 1,2,3-triazoloquinoxalin-4-one scaffold, further functionalized on its amide moiety mainly through alkylation reactions. However, these methodologies suffer from poor yields, the use of volatile and polluting solvents, highly toxic chemicals (*e.g.* diethyl oxalacetate for the triazole formation and dimethyl sulphate for the subsequent amide methylation step) and require the preparation of the appropriate starting compounds (*e.g.* the diamine or ethyl benzoyl- and 2-furoyl pyruvate sodium salts) (Biagi et al., 2002).

In 2012, Cai and Ding explored a strategy for the synthesis of [1,2,3]-triazolo [1,5-*a*]quinoxalin-4(5*H*)-ones starting from *N*-(2-iodophenyl)propiolamides, following a tandem azide-alkyne cycloaddition/Ullmann C-N coupling process. The exploration of the scope through the use of different alkyl and aryl substituents on the alkyne moieties (R_2_) as well as electron-donating and -withdrawing substituents (R_1_) on the 1-(2-iodoaryl) ring (Scheme 1C). However, these linear approaches are generally not convenient for scaffold diversification; moreover, no further functionalization steps were carried out on the amide functionality in the same paper (Yan et al., 2012).

Very recently, Li and coworkers reported a photoredox-catalyzed [3 + 2] cyclization reaction for the synthesis of [1,2,3]-triazolo-[1,5-*a*]quinoxalin-4(5*H*)-ones through quinoxalinones and hypervalent iodine (III) reagents, thus witnessing the renewed interest for innovative synthetic procedures towards this scaffold (Wen et al., 2022). Despite the simple proposed strategy for the preparation of 1*H*-quinoxalinones, starting from *o*-phenylenediamine and ethyl 2-oxoacetate and the subsequent alkylation with halogenoalkanes in DMF as solvent, it is important to highlight that the disclosed protocol required the preparation of the α-aryliodonio diazo compounds starting from ethyl diazoacetate (EDA) and bis(onio)-substituted aryliodine (III) salts or (diacetoxyiodo) benzene (Weiss et al., 1994; Xu et al., 2021). Furthermore, this method displays several drawbacks, lacking variability of substitutions on the triazoloquinoxaline scaffold, only reporting halo/methyl substitutions on the phenyl ring and envisaging the use of DMF and DCE as solvents. In addition, *N*-free quinoxalinone could not promote the reaction to generate the desirable product, making necessary the upstream *N*-alkylation step, thus affecting protocol versatility and product diversification (Scheme 1D).

**SCHEME 2:**
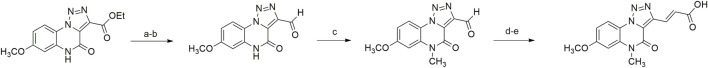
Previously reported approach for [1,2,3]-triazolo [1,5-*a*] quinoxalin-4(5*H*)-one derivatization. ^a^Reaction and conditions: **(A)** LiBH_4_, THF, 65°C; **(B)** Dess Martin reagent, CH_2_Cl_2_, rt, 84% over two steps; **(C)** MeI, K_2_CO_3_, DMF, 80°C, 15%; **(D)** MeO_2_CCH_2_P(O)(OMe)_2_, *n*-BuLi, THF, 0°C to rt; **(E)** LiOH, THF/MeOH/H_2_O, rt, 81% over two steps.

Only few examples of direct derivatization of the triazole esters have been previously reported, probably due to the low solubility of the deriving carboxylic acids and lengthy purification steps required; among them, it is reported conversion into aldehyde, without isolation of the alcohol intermediate and providing a long chain connected to the triazole ring with multiple synthetic steps using toxic reagents ^9^ (Scheme 2) (HongShen et al., 2009).

**SCHEME 3:**
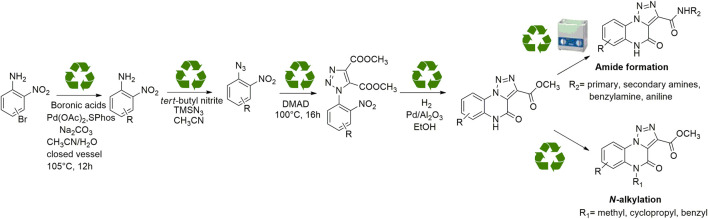
General scheme for the newly conceived sustainable approach for the synthesis and derivatization of [1,2,3]-triazolo [1,5-a] quinoxalin-4(5*H*)-ones.

For these reasons, the development of new methodologies for an easy access to this heterocyclic scaffold in a sustainable guise would be a nice and useful add-on to the currently limited armamentarium. As reported before, compared to the previous syntheses, we reasoned to plan a new ecofriendly methodology able to rejuvenate the chemical path toward [1, 2, 3]-triazolo [1,5-a]quinoxalin-4(5*H*)-one in a green and sustainable declination exploring for the first time on this scaffold a catalytic direct amidation and demonstrating the scope through the use of aliphatic, benzylic and aromatic amines. During our investigation, we used eco-sustainable reagents and catalysts for all the synthetic steps, performing reactions, whenever possible, through neat conditions, avoiding the hydrolysis of the esters and the pre-activation of the carboxylic acid with coupling agents and, thus, improving both yields and total atom economy of the synthesis (Scheme 3).

## 2 Results and discussion

### 2.1 Chemistry

In order to perform our 1,2,3-triazoloquinoxaline synthesis in a green declination and to expand the final library with focused structural modifications, our devised protocol started from in-house synthesized (through a sustainable Suzuki coupling protocol) (**2a-b**) or commercially available (**2c-i**) substituted 2-nitro anilines. Anilines were then converted into their corresponding 2-nitro azides (**3a-i**) using *tert*-butyl nitrite and trimethyl silyl azide that, compared to the reported procedures using sodium nitrite and sodium azide, could be considered as non-toxic and inert reagents (Barral et al., 2007) (Scheme 3). The resulting azides were then engaged in the click reaction with dimethyl acetylene dicarboxylate as the alkyne, without the need of catalysts or halogenated solvents providing the corresponding dimethyl carboxylate triazoles (**4a-i**) and avoiding the formation of previously described byproducts when using diethyl oxalacetate, ethyl benzoyl pyruvate and ethyl 2-furoyl pyruvate sodium salts. It is worth of note that for the generation of 1,4,5-trisubstituted 1,2,3-triazole moieties, only 2 references for the click reaction of 2-nitrophenyl azides and dimethyl acetylenedicarboxylate have been reported in literature, using copper nanoparticles immobilized on silk fibroin (Mirzaei et al., 2021) or regular stirring and microwave irradiation in dichloromethane (Souad et al., 2011). A relevant part of this project was then devoted to the exploration of different eco-compatible reaction conditions, using 2-nitrophenyl azide and dimethyl acetylene dicarboxylate as the starting materials in combination with different solvents, ionic liquids and catalysts, and also through the use of microwave and ultrasound bath (Table 1). When the reaction was performed in acetonitrile/water using copper sulphate and sodium ascorbate in order to generate Cu(I) required for the azyde/alkyne cycloaddition (Table 1, entry 1) a disappointing 4% yield of the desired compound was obtained. We then sought to explore ethanol both in the presence or not of Cu(I) (Table 1, entries 2 and 3), thus obtaining the triazole derivative in 44% and 40% yields, respectively. A further investigation using acetonitrile (Table 1, entry 4) and 2-Me-THF (Table 1, entry 5) for 12 h at reflux temperature, provided the desired compound in 28% and 61% yield, respectively. Surprisingly, when performed using 2-Me-THF as green solvent (Table 1, entry 6) under microwave condition, the reaction yield dropped to 17%. Also, the use of a ionic liquid as BMIM PF_6_ did not bring significant benefits, leading to the desired compound in 36% yield (Table 1, entry 7). We then sought to investigate the neat reaction conditions using microwave irradiation (entries 8 and 9), oil bath traditional heating (entry 10) and ultrasound bath (entries 11 and 12), monitoring the reaction through TLC. We found the best conditions driving a neat reaction at 100°C for 16 h (entry 10) with 80% yield.

**TABLE 1:** Optimization of reaction conditions for triazoles synthesis.

Entry	DMAD (eq)	Solvent	Catalyst	Temperature	Yield
1	1 eq	CH_3_CN/H_2_O (0.24M)10:1	CuSO_4_/sodium ascorbate	Rt	4%
2	1 eq	EtOH (0.2M)	-	reflux	44%
3	1 eq	EtOH (0.2M)	CuI 5% mol	reflux	40%
4	1 eq	CH_3_CN (0.2M)	-	reflux	28%
5	1 eq	2-Me-THF	-	reflux	61%
6	1 eq	2-Me-THF	-	MW; 100°C, 300W	17%
7	2 eq	0.25 eq BMIM PF_6_	-	MW; 100°C, 300W	36%
8	2 eq	neat	-	MW; 100°C, 300W	52%
9	2 eq	neat	-	MW; 120°C, 300W	54%
10	2 eq	neat	-	100°C	80%
11	2 eq	neat	-	ultrasound, rt	traces
12	2 eq	neat	-	ultrasound, 50 °C	traces

Once optimized the reaction conditions for the cycloaddition step, we then explored the reaction scope through the synthesis of differently substituted 1-aryl 1,2,3-triazole derivatives, bearing electron withdrawing, donating and biphenyl groups, with yields ranging from 40% to 98% (Table 2). Interestingly, only few of the synthesized triazoles were reported in literature, in lower yields when compared to our protocol; most of them were not previously described, highlighting the importance of this procedure also for the effective aryl triazole moiety generation.

**TABLE 2:** 1,2,4-Triazoles formation scope using optimized conditions.

Cmp	Triazoles	Yield (%)
**4a**	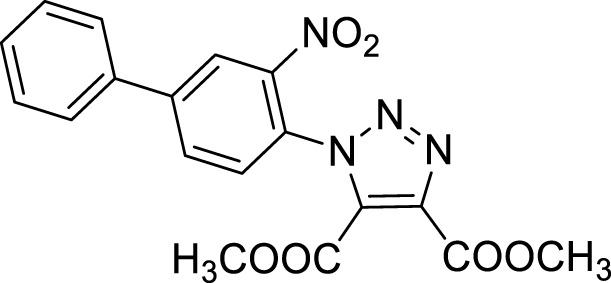	84
**4b**	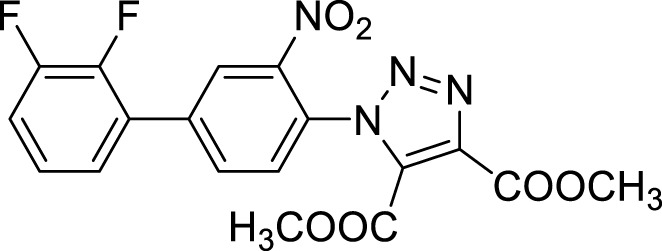	65
**4c**	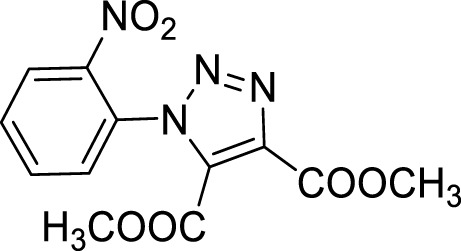	80^15^
**4d**	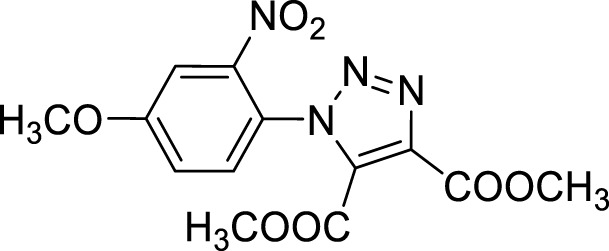	98
**4e**	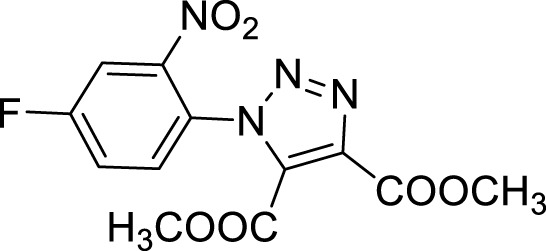	89
**4f**	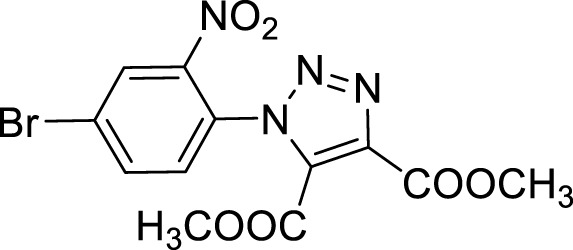	66
**4g**	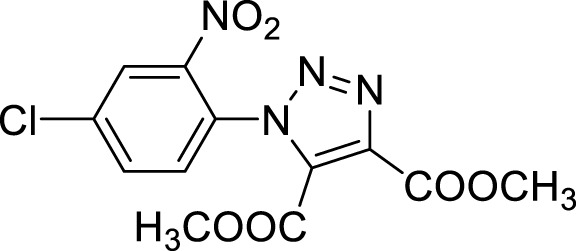	51
**4h**	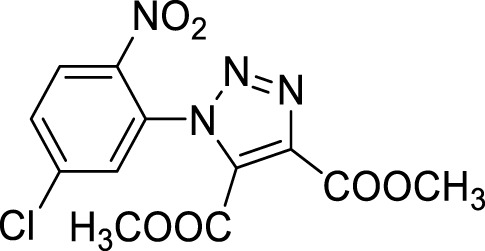	50
**4i**	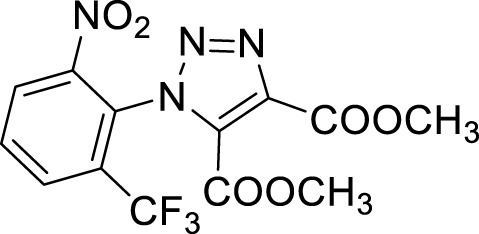	40

**SCHEME 4:**
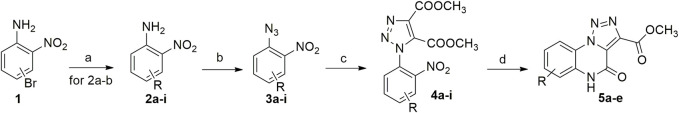
Eco-sustainable syntheses of triazoloquinoxalines. ^a^Reagents and conditions: **(A)** boronic acids, Pd(OAc)_2_, SPhos, Na_2_CO_3_, CH_3_CN/H_2_O (0.30 M; 3:1), 105°C; **(B)** *tert*-butyl nitrite, TMSN_3_, CH_3_CN, rt; **(C)** Dimethyl acetylenedicarboxylate; **(D)** H_2_, 5% loading Pd/Al_2_O_3_, rt.

Synthesized triazoles were then converted into the corresponding [1,2,3]-triazolo [1,5-*a*]quinoxalin-4(5*H*)-ones (**5a-e**) through nitro group reduction and following spontaneous cyclization, using hydrogen gas generator that produces hydrogen in a safe and convenient way, through electrolysis of water with a sustainable palladium on alumina catalyst 5% wt (Scheme 4). Reaction scope was also investigated, exploring different substituents on the phenyl ring, thus generating [1,2,3]-triazolo [1,5-*a*] quinoxalin-4(5*H*)-ones **5a-e**. As expected, when submitted to these reaction conditions, triazoles **4f, g, h**, only provided their de-halogenated counterpart **5c** (Table 3).

**TABLE 3:** Optimized conditions for the direct amidation of methyl 4-oxo-4,5-dihydro [1,2,3]triazolo [1,5-*a*]quinoxaline-3-carboxylate.

Entry	Amine	Solvent	Catalyst	Temperature/time	Yield
1	Benzylamine (1.3 eq)	Toluene	-	110°C, 12 h	quantitative
2	Benzylamine (1.3 eq)	Toluene	Cp_2_ZrCl_2_ 10% mol	110°C, 12 h	quantitative
3	Benzylamine (1.3 eq)	Toluene	CaI_2_ 10% mol	110 °C, 12 h	quantitative
4	Benzylamine (2.6 eq)	-	-	rt, 2 h	quantitative
5	Benzylamine (2.6 eq)	-	-	Ultrasound, 15′	quantitative
6	Cyclohexylamine (2.6 eq)	-	-	Ultrasound, 15′	97%
7	Pyrrolidine (2.6 eq)	-	-	Ultrasound, 15′	99%
8	Aniline (2.6 eq)	-	-	Ultrasound, 15′	Nd
9	Aniline (1.3 eq)	Toluene (4 M)	CaI_2_	110 °C, 12 h	traces
10	Aniline (1.3 eq)	Toluene (4 M)	Cp_2_ZrCl_2_ 10% mol	110 °C, 12 h	80%
11	Aniline (1.3 eq)	CPME	Cp_2_ZrCl_2_ 10% mol	110 °C, 12 h	81%

**SCHEME 5:**
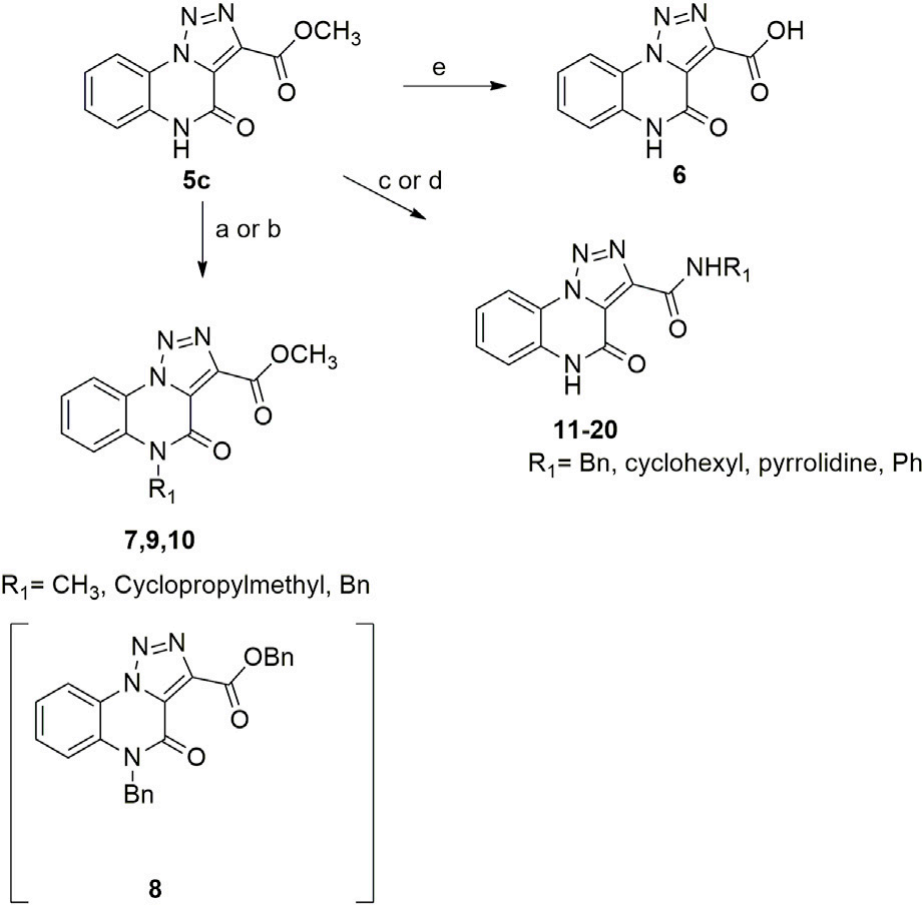
Hydrolysis, *N-* derivatization and direct amidation of [1,2,3]-triazolo [1,5-*a*]quinoxalin-4(5*H*)-one scaffold. ^a^Reagents and conditions: **(A)** dimethyl carbonate, K_2_CO_3_, closed vessel, 140°C, 16 h; **(B)** benzyl bromide or cyclopropyl methyl bromide, K_2_CO_3_, cyclopentylmethylether (0.1 M), 115°C, reflux, 12 h; **(C)** cyclohexylamine, pyrrolidine or benzyl amines, ultrasound bath, rt, 15'; **(D)** aniline, cyclopentylmethylether (4M), Cp_2_ZrCl_2_ 10 mol%, 110°C, 12 h; **(E)** NaOH, MeOH/H_2_O (0.25 M, 3:1), 1.5 h.

With our [1,2,3]-triazolo [1,5-*a*] quinoxalin-4(5*H*)-ones in hand, we started our campaign for its sustainable derivatization. First of all, we performed a simple alkaline hydrolysis of the ester group of compound **5c** with sodium hydroxide in a mixture of methanol and water, which provided the corresponding carboxylic acid derivative **6** (Scheme 5).

Regarding the *N*-methylation step for the [1,2,3]-triazolo [1,5-*a*] quinoxalin-4(5*H*)-one scaffold, the reported protocol encompassed the use of dimethyl sulfate and iodomethane as methylating agents, obtaining the *N*-methylated derivative with yields of 64% and 15%, respectively ^13^. In our quest for a more sustainable protocol, we decided to carry on compound **5c** using the greener dimethyl carbonate, taking advantage of its dual role as both solvent and electrophilic reagent (Fiorani et al., 2018), and potassium carbonate as the base in a closed vessel at 140 °C for 12 h. This protocol allowed to obtain derivative **7** with a satisfying 78% yield (Scheme 5).

We then shifted to interrogate a benzyl substitution on the endocyclic amide nitrogen. After a small investigation of reaction conditions, we carried out the reaction in the presence of benzyl bromide and potassium carbonate, using cyclopentyl methyl ether (CPME), a versatile eco-friendly solvent with high boiling point and a low peroxide formation rate (Watanabe et al., 2007), thus almost quantitatively obtaining the desired compound **9** with only traces of the disubstituted derivative **8** (Scheme 5). Noteworthy, the same reaction carried out using dimethylformamide as the solvent, gave the *N*-benzylated compound **9** in 34% yield due to the concomitant formation of higher amounts of the disubstituted counterpart **8** (40% yield).

Likewise, compound **10** was obtained using cyclopropyl methyl bromide and potassium carbonate in CPME (Scheme 5).

Our further efforts were devoted to the functionalization of the ester moiety on the 1,2,3-triazole ring. To this aim, we explored an unprecedented direct amidation reaction on this scaffold (Scheme 5 and Table 3). For this protocol, we first treated compound **5c** with benzylamine using toluene as the solvent. The reaction proceeded quantitatively both in absence (Table 3, entry 1) and in the presence of sustainable amidation catalysts such as dichlorobis (cyclopentadienyl)zirconium (Cp_2_ZrCl_2_) (Table 3, entry 2) and calcium iodide (Table 3, entry 3). Gratifyingly, the reaction provided a quantitative outcome even under neat conditions at room temperature (Table 1, entry 4). Finally, we found the best condition by direct irradiation with an ultrasound bath for 15 min without any catalyst (Table 3, entry 5) and we applied the same conditions to both an aliphatic primary amine (cyclohexylamine, Table 3, entry 6) and a secondary amine (pyrrolidine, Table 3, entry 7) obtaining quantitative conversions for all the starting materials, thus confirming the broad scope of this amidation protocol. However, when trying to extend the procedure to anilines we did not observe product formation (Table 3, entry 8). Our previous results with eco-sustainable catalysts prompted us to test the coupling with aniline using calcium iodide (Table 3, entry 9) and Cp_2_ZrCl_2_ dichlorobis (cyclopentadienyl) zirconium (Table 3, entry 10) in toluene at 110°C overnight. Only when using Cp_2_ZrCl_2_ as the catalyst, we were able to obtain the desired benzamide in 80% yield (Table 3, entry 10). Gratifyingly, replacement of toluene with CPME, was successful for the conversion into amide with a sustainable protocol (Table 3, entry 11) and in 81% yield.

The full library of 20 derivatives synthesized within this study is reported in Table 4.

**TABLE 4:** Full library of synthesized [1,2,3]-triazolo [1,5-a] quinoxalin-4(5*H*)-ones within this work.

Cmp	[1,2,3]-triazolo [1,5-a] quinoxalin-4(5*H*)-ones	Yield
**5a**	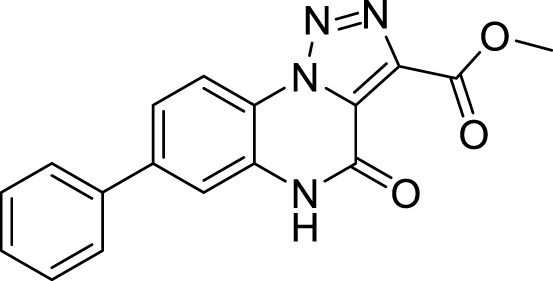	20%
**5b**	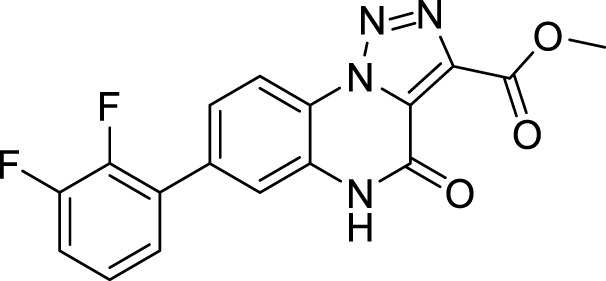	27%
**5c**	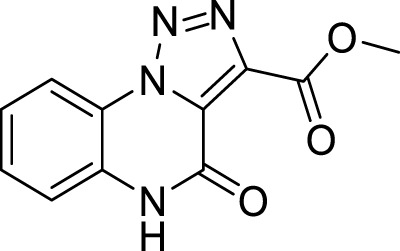	65%
**5d**	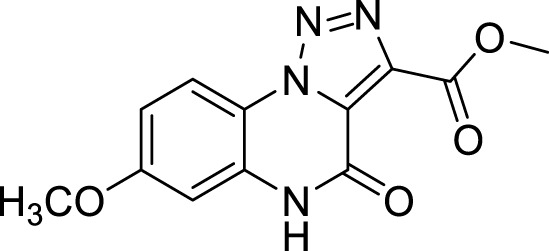	56%
**5e**	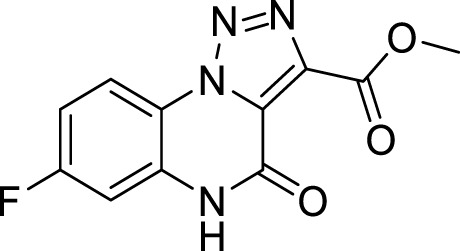	35%
**6**	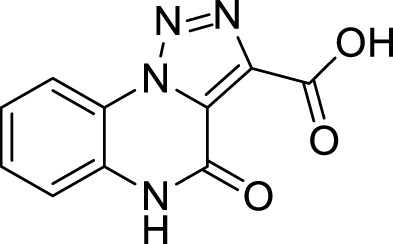	quantitative
**7**	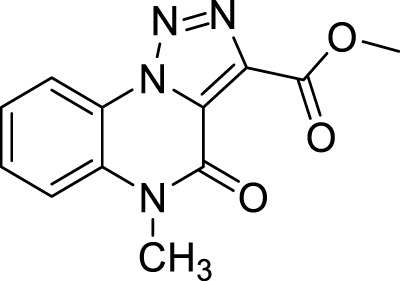	78%
**8**	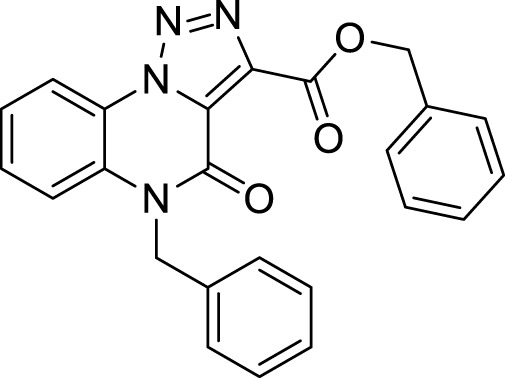	traces
**9**	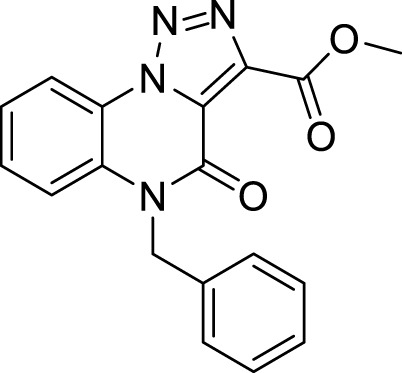	quantitative
**10**	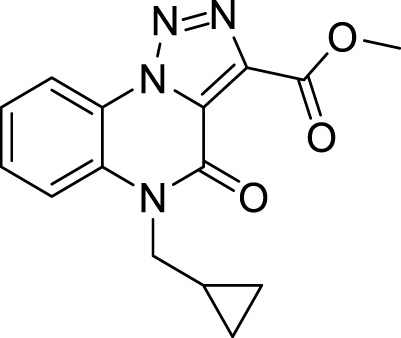	86%
**11**	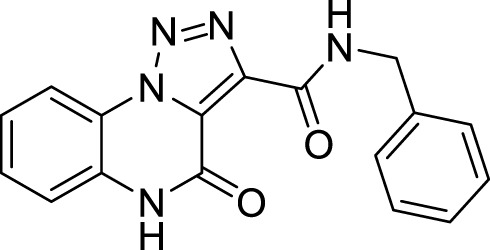	quantitative
**12**	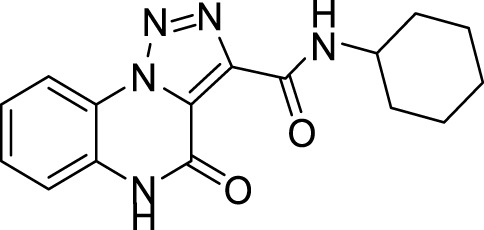	97%
**13**	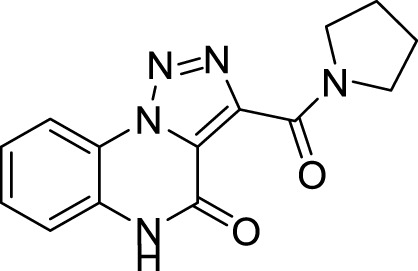	99%
**14**	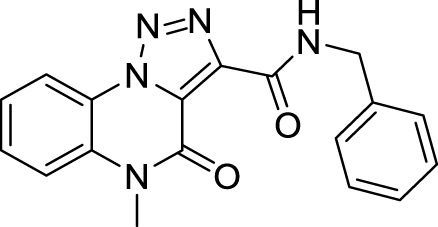	94%
**15**	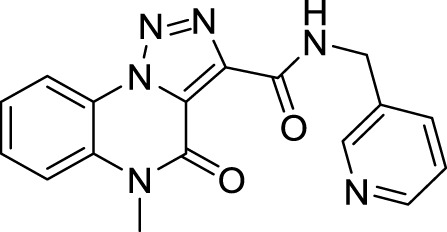	95%
**16**	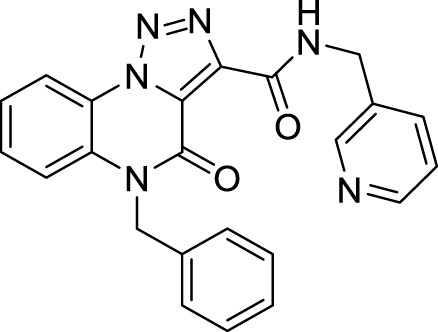	96%
**17**	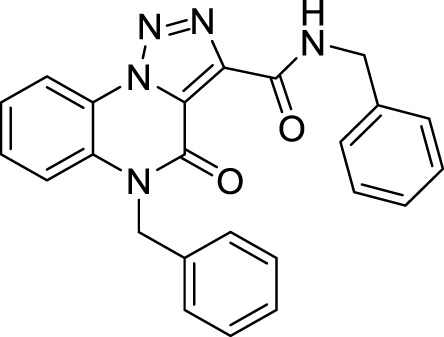	quantitative
**18**	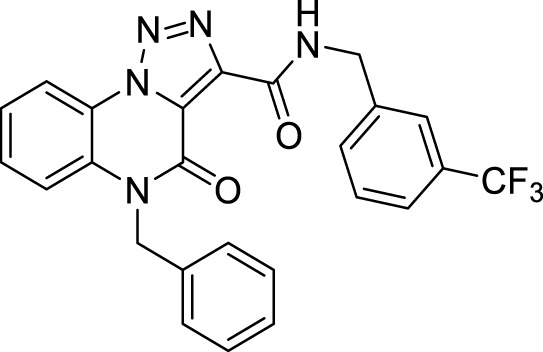	89%
**19**	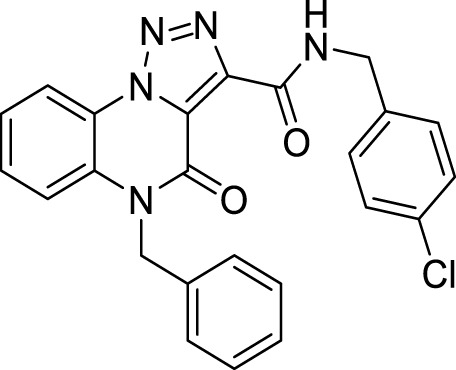	93%
**20**	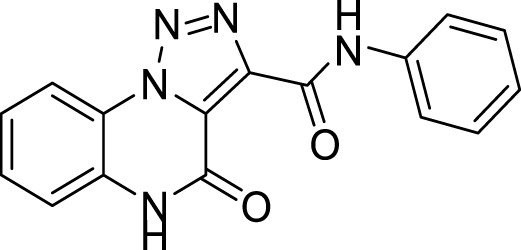	81%

### 2.2 Biological evaluation as antimicrobial agents

According to previous reports highlighting antibacterial properties for some triazoloquinoxalinone derivatives, we decided to perform a preliminary biological investigation for evaluating the antimicrobial potential in multiple bacterial and fungal species of our newly synthesized derivatives. Polymicrobial infections, caused by a simultaneous infection of viruses, bacteria, fungi, and parasites, represent an emerging and quickly increasing phenomenon, due to the possibility of one pathogen to predispose the host to colonization by other pathogens. These infections are more tolerant to antibiotic therapy, thus rendering necessary the search for broad-spectrum antimicrobials, which would display several advantages with respect to a therapy with multiple antibiotics. Another relevant issue to be taken into account when dealing with polymicrobial infections is the biofilm formation, which can be reasonably considered as a resistance mechanism adopted by some bacterial species, able to generate a self-produced extracellular polymeric substance (EPS) to form a matrix. Biofilm provides a survival strategy and protection against antibiotics, acting as a reservoir for the cellular exchange of plasmids encoding for resistance to antibiotics. In this context, the discovery of new compounds able to counteract both Gram-positive and Gram-negative bacteria or yeasts, while reducing at the same time biofilm formation, is of crucial importance.

**FIGURE 1:**
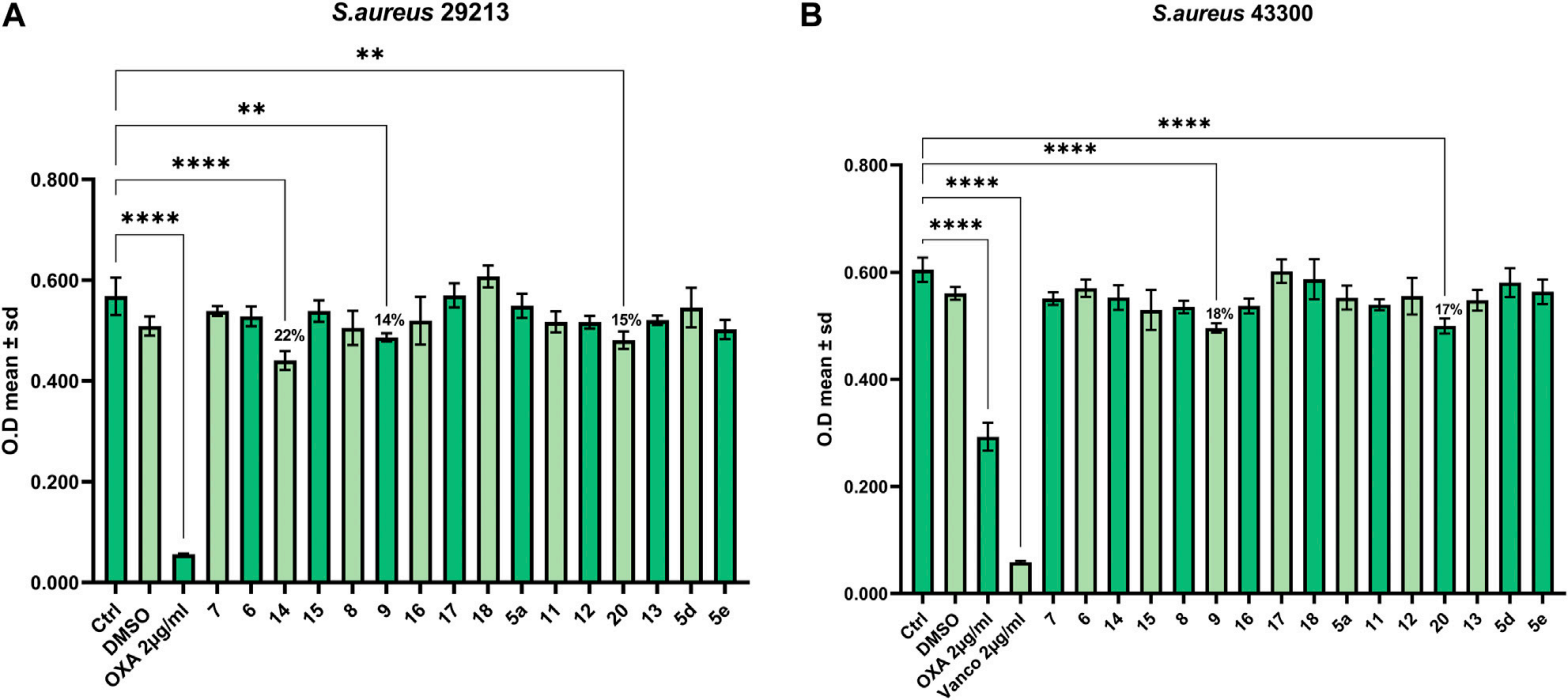
Antibacterial effect against Gram-positive **(A)** *S. aureus* ATCC 29213 and **(B)** *S. aureus* ATCC 43300.

**FIGURE 2:**
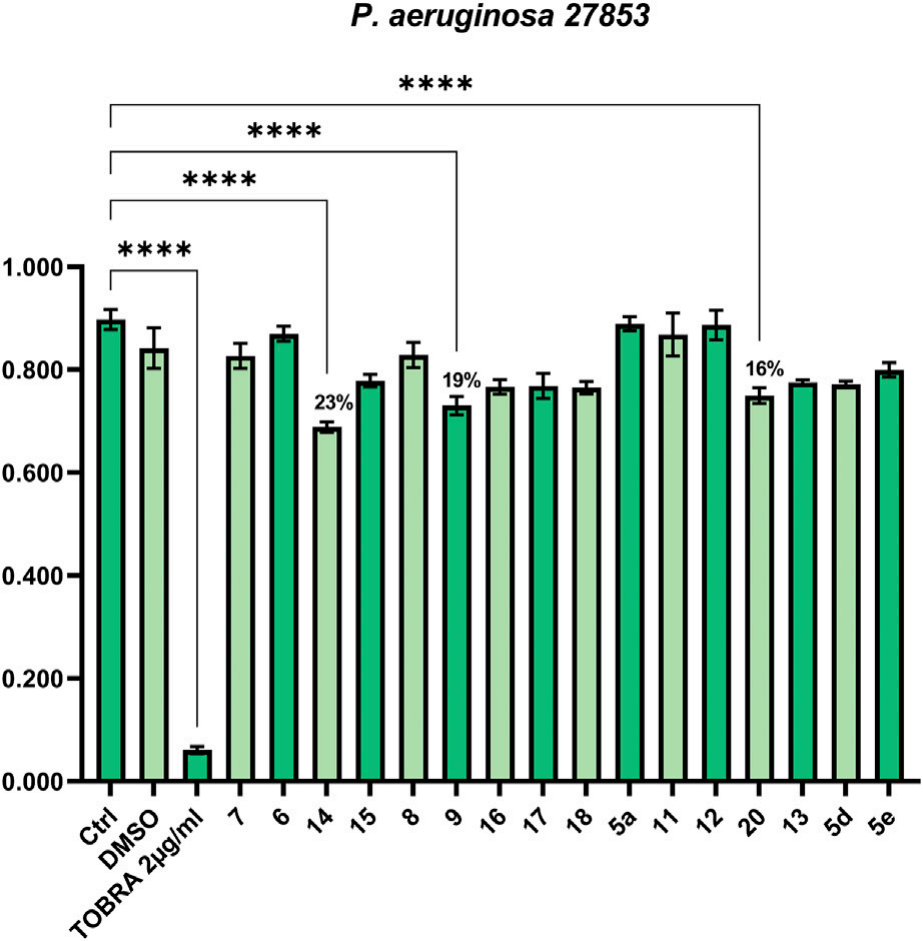
Antibacterial effect against Gram-negative *P. aeruginosa* 27853.

To assess the antimicrobial properties of compounds **5a**, **5d**, **5e**, **6-9**, **11-18**, and **20**, the minimal inhibitory concentrations (MIC) were determined by broth microdilution assay. The assay was preliminarily performed against different pathogenic bacteria strains to verify if the compounds were able to inhibit cell growth. The examined strains were two Gram-positive, *S. aureus* ATCC 29213 and *S. aureus* ATCC 43300 (MRSA), and three Gram-negative, *P. aeruginosa* ATCC 27853, *K. pneumoniae* ATCC 13883, and *K. pneumonia* ATCC BAA-1705. The antibiotics OXA, VAN, TOB, and IPM were used as a control against *S. aureus*, *P. aeruginosa*, and *K. pneumoniae*, according to European Committee on Antimicrobial Susceptibility Testing (EUCAST version 12.0, 2022). As reported in Figures 1, 2 none of the compounds was able to produce a MIC value at the tested concentrations (up to 100 μM). However, three compounds were able to slightly affect the growth of the microorganisms studied. Compounds **9** (SL69) and **20** (QNX55) were able to reduce the growth of both *S. aureus* ATCC strains at the highest concentration with a percentage of reduction ranging between 15%-18% (Figures 1A, B). Compound **14** (SL66) was able to reduce by 22% also the growth of *S. aureus* ATCC 29213. The same compounds **9**, **14** and **20** (SL66, SL69, QNX55) were able to affect the growth of *P. aeruginosa* (Figure 2), but not *K. pneumoniae* (data not shown). The best activity was observed with compound **14** (SL66), able to cause about a 23% reduction in *P. aeruginosa* cell growth.

**FIGURE 3:**
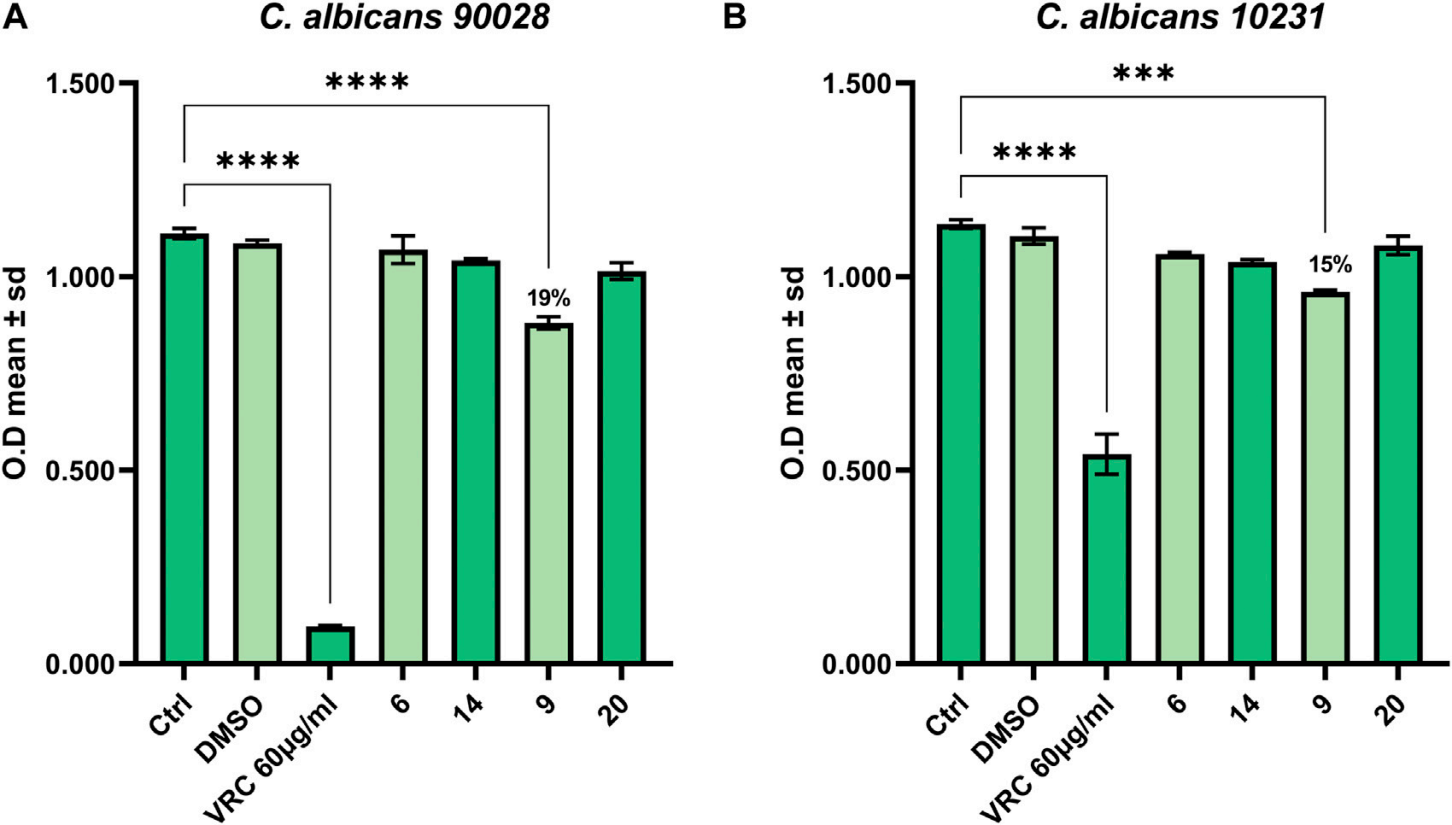
Antifungal activity against **(A)** *C. albicans* 90028 and **(B)** *C. albicans* azole-resistant 10231.

The compounds showing the best results in the antimicrobial assay were also tested for their antifungal properties against the yeast *C. albicans*. Among the three compounds, only compound **9** (SL69) was able to contrast the growth of both strains of *C. albicans*, the control strain and the azole-resistant one (Figures 3A, B).

**FIGURE 4:**
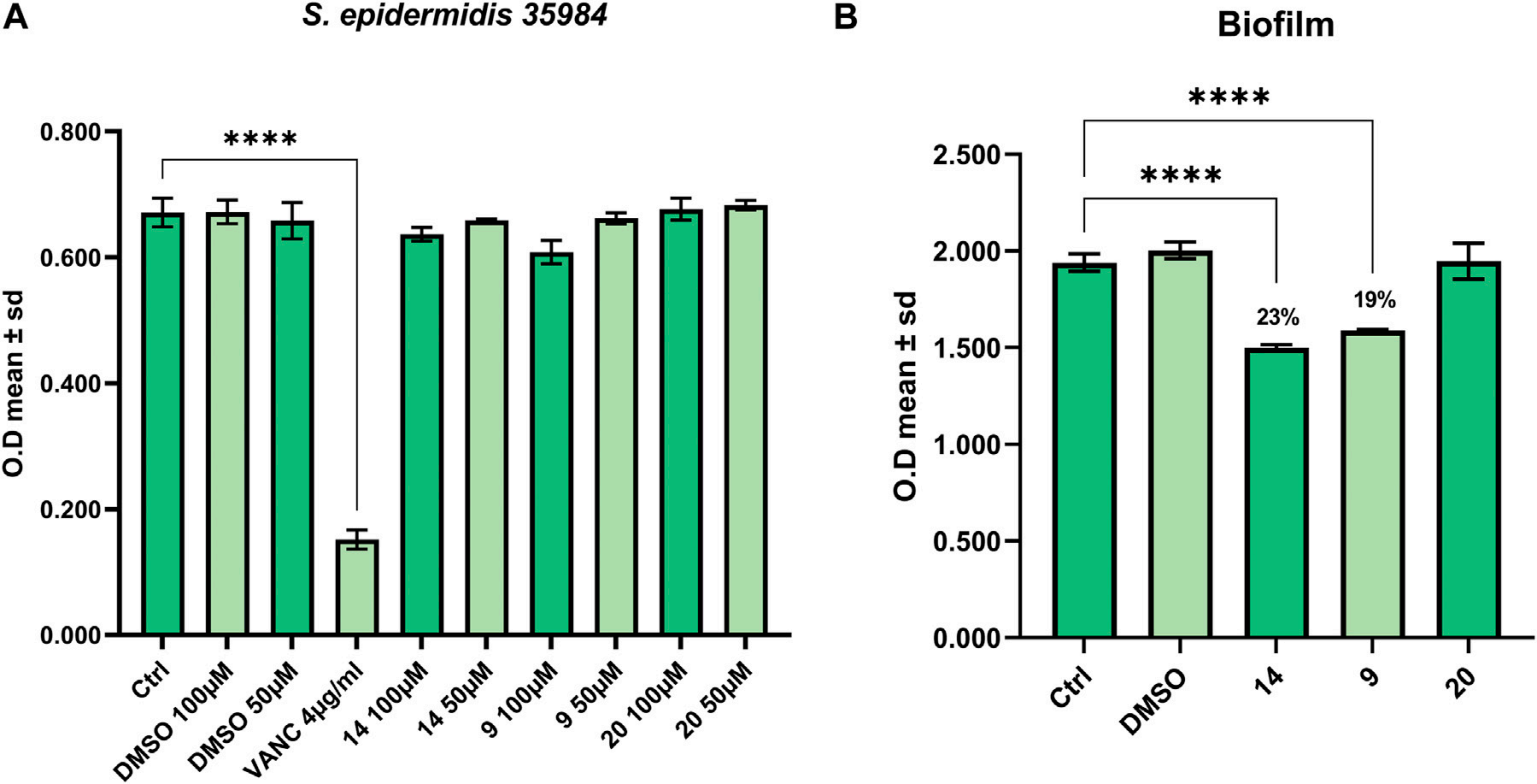
Antibacterial activity against **(A)** S. *Epidermidis* 35984 and **(B)** inhibition of biofilm formation.

Finally, we tested the activity of derivatives **9**, **14** and **20** (L66, SL69, and QNX55) to affect the growth of *S. epidermidis* ATCC 35984 and to contrast its ability to form biofilm (Vollaro et al., 2019; Alfano et al., 2021). As shown in Figure 4A only compound **7** (SL69) slightly affected the growth of *S. epidermidis* at the highest concentration (100 μM), while at 50 μM we did not observe cell growth inhibition. Consequently, since 50 μM compound **9**, **14** and **20** (SL66, SL69, and QNX55) did not produce a bacteriostatic effect the latter was chosen as the concentration useful to perform the biofilm assay. As reported in Figure 4B, compounds **9** and **14** (SL66 and SL69) were both able to contrast the biofilm formation by 18% and 23%, respectively.

The results here obtained are encouraging since three out of 16 compounds displayed a limited antimicrobial property, but compound **9** (SL69) reduced the growth of different bacterial strains and the yeast *Candida*, also affecting biofilm formation in *S. epidermidis*. Taken together, these data foster further optimization of the compounds and the synthetic protocol herein developed guarantees straightforward and sustainable scaffold morphing and derivatization.

## 3 Materials and methods

### 3.1 Chemistry

#### 3.1.1 General methods

Commercially available reagents and solvents were used without further purification. When necessary, the reactions were performed in oven-dried glassware under a positive pressure of dry nitrogen. Melting points were determined in open glass capillaries and are uncorrected. All the compounds were characterized by ^1^H and ^13^C APT NMR that were recorded at a Bruker Avance NEO instruments (400 and 700 MHz). Mass spectra of final products were performed on LTQ Orbitrap XL™Fourier transform mass spectrometer (FTMS) equipped with an ESI ION MAX™ (Thermo Fisher, San José, USA) source operating in positive mode. The spectra were recorded by infusion into the ESI source using MeOH as the solvent. Chemical shifts (δ) are reported in part per million (ppm) relative to the residual solvent peak. Column chromatography was performed on silica gel (70–230 mesh ASTM) using the reported eluents. Thin layer chromatography (TLC) was carried out on 5 × 20 cm plates with a layer thickness of 0.25 mm (Silica gel 60 F254). The purity of all compounds was confirmed by NMR and analytic HPLC-UV as ε 92%-95%.

#### 3.1.2 General preparation of substituted anilines

A microwave vial was charged under argon with the corresponding halo-derivative (0.92 mmol), the corresponding phenylboronic acid (1.2 equiv.), Pd(OAc)_2_ (10% mol), Sphos (20%mol) Na_2_CO_3_ (3.0 equiv.) and a mixture of acetonitrile:water (0.30M; 2:1). The vial was capped properly, flushed with argon and heated to 105 °C until complete conversion of the starting material. After it was cooled, the reaction mixture was concentrated under vacuum. The crude residue was diluted in water. The organic phase was extracted 3 times with EtOAc. The organic layers were combined, washed with brine, dried over Na_2_SO_4_, filtered, concentrated and purified by silica gel chromatography column, eluting with the appropriate *n*-hexane: ethylacetate mixture.

**3-Nitrobiphenyl-4-amine (2a)**. The crude material was purified by column chromatography (*n-*hexane/EtOAc 80:20) to give the product as a red-brown crystals (172 mg, 87% yield).

^1^H NMR (DMSO-d_6_, 400 MHz): δ 8.22 (s, 1H), δ 7.79 (d, *J =* 8.8 Hz, 1H), δ 7.63 (d, *J =* 8 Hz, 2H), δ 7.54 (s, 2H), δ 7.44 (t, *J =* 7.6 Hz, 2H), δ 7.33 (t, *J =* 7.2 Hz, 1H), δ 7.13 (d, *J =* 8.8 Hz, 1H). ^13^C APT (DMSO-d_6_; 176 MHz): δ 146.01, 138.74, 134,73, 130.91, 129.48, 127.83, 127.50, 126.23, 122,84,120.49.

**2′,3′-Difluoro-3-nitrobiphenyl-4-amine (2b).** ^1^H NMR (DMSO-d_6_, 400 MHz): δ 8.18 (s, 1H), δ 7.67-7.64 (m, 3H), δ 7.43-7.36 (m, 2H), δ 7.31-7.25 (m, 1H), δ 7.14 (d, *J =* 9.2 Hz, 1H). ^13^C APT (DMSO-d_6_; 176 MHz): δ 151.4, 150.1, 148.2, 146,8, 146.42, 136.30, 130.54, 129.18, 125.8, 125.6, 125.5, 121.16, 120.22, 116.6.

#### 3.1.3 General preparation of azides

**4-Azido-3-nitrobiphenyl (3a).** The crude material was purified by column chromatography (*n*-hexane/EtOAc 98:1) to give the product as an orange solid (97% yield).

^1^H NMR (CDCl_3_, 400 MHz): δ 8.1 (s, 1H), δ 7.76 (d, *J =* 8.8 Hz, 1H), δ 7.51 (d, *J =* 8.0 Hz, 2H), δ 7.41 (t, *J =* 7.6 Hz, 2H) δ, 7.37-7.32 (m, 2H). ^13^C APT (CDCl_3_; 176 MHz): δ 138.52, 137.69, 133.54, 132. 28, 129.26, 128.61, 126.85, 124.37, 121.26.

**4′-Azido-2,3-difluoro-3′-nitrobiphenyl (3b).** The crude material was purified by column chromatography (*n-*hexane/EtOAc 98:1) to give the product as an orange solid (90% yield). ^1^HNMR (CDCl_3_, 400 MHz): δ 8.06 (s, 1H), δ 7.74 (d, *J =* 8.4 Hz, 1H) δ,7.36 (d, *J =* 8.4 Hz, 1H), δ 7.17-7.13 (m, 3H). ^13^C APT (CDCl_3_; 176 MHz): δ 151.89, 150.46, 148.76, 147.25, 140.86, 134.59, 134.26, 131.77, 127.85, 126.31, 124.78, 121.07, 118.19, 117.49.

**1-Azido-2-nitrobenzene (3c).** 2-Nitroaniline (280 mg, 2.02 mmol) was dissolved in acetonitrile (4 ml) in a 25 ml round-bottomed flask and cooled to 0°C in an ice bath. *t*-BuONO (312 mg, 360 *μ*L, 3.03 mmol) was then added followed by TMSN_3_ (279 mg, 322 *μ*L, 2.42 mmol) dropwise. The resulting solution was stirred at room temperature for 1 h. The reaction mixture was concentrated under vacuum and the crude product was purified by silica gel chromatography (*n*-hexane) to give the product, as a pale yellow solid (327 mg, 98%).

^1^H NMR (CDCl_3_, 400 MHz): δ 7.87 (d, *J =* 8.0 Hz, 1H), δ 7.55 (t, *J =* 8.0 Hz, 1H), δ 7.27 (d, *J =* 8.8 Hz, 1H), δ 7.19 (t, *J =* 8.4, 1H). ^13^C APT (CDCl_3_; 176 MHz): δ 140.92, 134.84, 126.13, 124.93, 120.79.

**1-Azido-4-methoxy-2-nitrobenzene (3d).** The crude material was purified by column chromatography (*n*-hexane/EtOAc 98:2) to give the product as a yellow solid (265 mg, 82% yield). ^1^H NMR (CDCl_3_, 400 MHz): δ 7.39 (s, 1H), δ 7.17 (d, 1H, *J =* 8.8 Hz), δ 7.12-7.09 (m, 1H), δ 3.79 (s, 3H). ^13^C APT (CDCl_3_; 176 MHz): δ 156.49, 141.11, 127.22, 121.95, 121.26, 110.14, 56.12.

**1-Azido-4-fluoro-2-nitrobenzene (3e).** The crude material was purified by column chromatography (*n*-hexane/EtOAc 98:1) to give the product as an orange oil (269 mg, 89% yield).

^1^H NMR (CDCl_3_, 400 MHz): δ 7.64-7.62 (m, 1H), δ 7.32-7.23 (m, 2H). ^13^C APT (CDCl_3_; 176 MHz): δ 159.07, 157.65, 131.06, 122.32, 121.68/121.55, 113.66/113.50.

**1-Azido-4-bromo-2-nitrobenzene (3f).** The crude material was purified by column chromatography (*n*-hexane/EtOAc 99:1) to give the product as a yellow solid (360 mg, 89% yield).

^1^H NMR (CDCl_3_, 400 MHz): δ 8.01 (s, 1H), δ 7.65 (d, *J =* 8.4 Hz, 1H), δ 7.15 (d, *J =* 8.8 Hz, 1H).

^13^C APT (CDCl_3_; 176 MHz): δ 141.08, 136.91, 134.05, 128.96, 122.20, 117.15.

**1-Azido-4-chloro-2-nitrobenzene (3g).** The crude material was purified by column chromatography (*n*-hexane/EtOAc 98:1) to give the product as a yellow solid (96% yield).

^1^H NMR (CDCl_3_, 400 MHz): δ 7.97 (s, 1H),7.61 (d, *J =* 8.4, 1H), δ 7.30 (d, *J =* 8.4 Hz, 1H). ^13^C APT (CDCl_3_; 176 MHz): δ 134.04, 133.54, 130.30, 126.14, 121.95.

**2-Azido-4-chloro-1-nitrobenzene (3h).** The crude material was purified by column chromatography (*n*-hexane/EtOAc 98:1) to give the product as a yellow solid (94% yield).

^1^H NMR (CDCl_3_, 400 MHz): δ 7.95 (d, *J =* 8.8 Hz, 1H), δ 7.34 (s, 1H), δ 7.25 (d, *J =* 8.8, 1H). ^13^C APT (CDCl_3_; 176 MHz): δ 140.42, 136.40, 127.45, 125.19, 120.89.

#### 3.1.4 General preparation of triazoles

Dimethyl 1-(3-nitrobiphenyl-4-yl)-1*H*-1,2,3-triazole- 4, 5-dicarboxylate (4a).

^1^H NMR (CDCl_3_, 400 MHz): δ 8.42 (s, 1H), δ 7.96 (d, *J =* 8.0 Hz, 1H), δ 7.62-7.56 (m, 3H), δ 7.50-7.42 (m, 3H), δ 3.97 (s, 3H), δ 3.81 (s, 3H). ^13^C APT (CDCl_3_; 176 MHz): δ 160.07, 158.10, 145.70, 144.57, 139.81, 137.02, 132.26, 132.03, 130.23, 129.59, 129.49, 127.89, 127.30, 124.14, 53.68, 53.00.

**Dimethyl 1-(2′, 3′-difluoro-3-nitrobiphenyl-4-yl)-1*H*-1, 2, 3-triazole-4, 5-dicarboxylate (4b).** Sticky solid, 65% yield. ^1^H NMR (CDCl_3_, 400 MHz): δ 8.40-8.39 (m, 1H), δ 7.97-7.94 (m, 1H), δ 7.61 (d, *J =* 8.0 Hz, 1H), δ 7.27-7.21 (m, 3H), δ 3.98 (s, 3H), δ 3.82 (s, 3H). ^13^C APT (CDCl_3_; 176 MHz): δ 160.01, 158.03, 151.92 (151.85), 150.50 (150.43), 148.83 (148.75), 147.40 (147.32), 144.39, 139.89, 139.10, 134.42, 131.95, 130.13, 128.79, 127.22 (127.17), 126.06 (126.05), 125.10 (125.07,125.02, 125.00), 118.61, 118.52, 53.72, 53.04.

**Dimethyl 1-(2-nitrophenyl)-1*H*-1,2,3-triazole-4,5-dicarboxylate (4c).** Dimethyl acetylene dicarboxylate (2 equivalents) was then added to the 1-azido-2-nitrobenzene and the reaction was stirred 12 h at 85°C. The product was obtained after silica gel chromatography column (7:3 *n-*hexane/Ethyl acetate). ^1^H NMR (CDCl_3_, 400 MHz): δ 8.24 (d, *J =* 7.6 Hz, 1H), δ 7.82-7.72 (m, 2H), δ 7.52 (d, *J =* 7.6 Hz, 1H), δ 3.97 (s, 3H), δ 3.78 (s, 3H). ^13^C APT (CDCl_3_; 176 MHz): δ 160.04, 157.96, 144.37, 139.80, 134.37, 132.15, 131.98, 129.91, 129.43, 125.86, 53.63, 52.99.

Dimethyl 1-(4-methoxy-2-nitrophenyl)-1*H*-1,2,3-triazole-4,5-dicarboxylate (4d).

^1^H NMR (CDCl_3_, 400 MHz): δ 7.70 (s, 1H), δ 7.41 (d, *J =* 8.8 Hz, 1H), δ 7.24-7.21 (m, 1H), δ 3.96 (s, 3H), δ 3.91 (s, 3H), δ 3.78 (s, 3H). ^13^C APT (CDCl_3_; 176 MHz): δ 161.72, 160.11, 158.14, 145.18, 139.60, 132.20, 130.87, 121.69, 119,53, 110.96, 56.47, 53.59, 52.93.

Dimethyl 1-(4-fluoro-2-nitrophenyl)-1*H*-1, 2, 3-triazole-4, 5-dicarboxylate (4e).

^1^H NMR (CDCl_3_, 400 MHz): δ 7.97-7.95 (m, 1H), δ 7.56-7.47 (m, 2H), δ 3.97 (s, 3H), δ 3.80 (s, 3H). ^13^C APT (CDCl_3_; 176 MHz): δ 163.85, 162.38, 159.94, 157.93, 145.26, 139,92, 131.90, 131.78, 125.64, 121.54, 121.42, 114.03, 113.87, 53.70, 53.04.

Dimethyl 1-(4-bromo-2-nitrophenyl)-1*H*-1, 2, 3-triazole-4, 5-dicarboxylate (4f).

^1^H NMR (CDCl_3_, 400 MHz): δ 8.36 (s. 1H), δ 7.91 (d, *J =* 8.4 Hz, 1H), δ 7.40 (d, *J =* 8.4 Hz, 1H), δ 3.96 (s, 3H), δ 3.80 (s, 3H). ^13^C APT (CDCl_3_; 176 MHz): δ 159.88, 157.89, 144.54, 139.98, 137.41, 131.77, 131.03, 128.99, 128.35, 125.92, 53.72, 53.02.

**Dimethyl 1-(4-chloro-2-nitrophenyl)-1*H*-1, 2, 3-triazole-4, 5-dicarboxylate (4g).** Yellow solid, 51% yield. ^1^H NMR (CDCl_3_, 400 MHz): δ 8.31 (s, 1H), δ 7.85 (dd, 1H, *J =* 8.4 and 2.4 Hz), δ 7.57 (d, 1H, *J =* 8.4 Hz), δ 4.06 (s, 3H), δ 3.89 (s, 3H). ^13^C APT (CDCl_3_; 176 MHz): δ 159.91, 157.91, 144.59, 139.97, 138.40, 134.35, 131.81, 130.92, 127.86, 126.15, 53.74, 53.06.

**Dimethyl 1-(5-chloro-2-nitrophenyl)-1*H*-1, 2, 3-triazole-4, 5-dicarboxylate (4h).** Yellow solid, 50% yield. ^1^H NMR (CDCl_3_, 400 MHz): δ 8.29 (d, 1H, *J =* 8.8 Hz), δ 7.80 (dd, 1H, *J =* 8.8 Hz and 2.0 Hz), δ 7.62 (s, 1H), δ 4.06 (s, 3H), δ 3.90 (s, 3H). ^13^C APT (CDCl_3_; 176 MHz): δ 159.86, 157.81, 142. 59, 140.85, 139.96, 132.14, 131.75, 130.48, 130.13, 126.98, 53.75, 53.08.

**Dimethyl 1-(2-nitro-6-(trifluoromethyl)phenyl**)**-1*H*-1, 2, 3-triazole-4, 5-dicarboxylate (4i).** Light yellow oil, 40% yield. ^1^H NMR (CDCl_3_, 400 MHz): δ 8.42 (d, 1H, *J =* 8.4 Hz), δ 8.11 (d, 1H, *J =* 7.6 Hz), δ 7.92 (t, 1H, *J =* 8.0 Hz), δ 3.98 (s, 3H), δ 3.79 (s, 3H).

^13^C APT (CDCl_3_; 176 MHz): δ 159.72, 157,63, 146,10, 139.57, 132.57, 131.94, 129.89. 129.23, 128.06, 122.24, 120.68, 53.62, 53.07.

#### 3.1.5 General procedure for [1, 2, 3]-triazolo [1,5-*a*]quinoxalin-4(5*H*)-one scaffold

To a solution of the corresponding triazole diester in EtOH (0.036M), 10% Pd/Al_2_O_3_ (5% loading) was added and the mixture was hydrogenated at room temperature overnight. Ethanol was evaporated and the crude was purified by silica gel chromatography column (dichloromethane/methanol).

**Methyl 4-oxo-7-phenyl-4,5-dihydro-[1,2,3]triazolo[1,5-*a*]quinoxaline-3-carboxylate (5a).** White solid, 20% yield. ^1^H NMR (DMSO-d_6_, 400 MHz): δ 12.32 (s, 1H), δ 8.45 (d, *J =* 8.8 Hz, 1H), δ 7.72-7.68 (m, 4H), δ 7.55 (t, *J =* 7.6 Hz, 2H), δ 7.47 (t, *J =* 6.8 Hz, 1H), δ 3.95 (s, 3H). ^13^C APT (DMSO-d_6_; 176 MHz): δ 160.70, 152.69, 142.09, 139.06, 138.08, 130.65, 129.77, 128.93, 127.60, 127.32, 122.77. 120.49, 117.16, 114.75, 53.02. ESI-MS (m/z): 321.2 [M + H]^+^, 343.1 [M + Na]^+^, 359.2 [M + K] ^+^

**Methyl 7-(2,3-difluorophenyl)-4-oxo-4,5-dihydro-[1,2,3]triazolo[1,5-*a*]quinoxaline-3-carboxylate (5b).** White solid, 27% yield. ^1^H NMR (DMSO-d_6_, 400 MHz): δ 12.35 (s, 1H), δ 8.50 (d, *J =* 8.4 Hz, 1H), δ 7.63-7.61 (m, 2H), δ 7.58-7.51 (m, 1H), δ 7.46-7.36 (m, 2H), δ 3.95 (s, 3H). ^13^C APT (DMSO-d_6_; 176 MHz): δ 160.65, 152.64,151.39, 149.99, 148.19, 146.82, 138, 11, 135.53, 130. 33, 129.35, 127.84, 126.24, 125.99, 124.66, 121.04, 118.03, 117.93, 117.07, 53.04. ESI-MS (m/z): 357.2 [M + H]^+^, 379.2 [M + Na]^+^, 395.3 [M + K] ^+^

**Methyl 4-oxo-4, 5-dihydro-[1, 2, 3]triazolo[1,5-*a*]quinoxaline-3-carboxylate (5c).** White solid, 65% yield. ^1^H NMR (DMSO-d_6_, 400 MHz): δ 12.18 (s, 1H), δ 8.38 (d, *J =* 8.0 Hz, 1H), δ 7.50 (t, *J =* 7.6 Hz, 1H), δ 7.46 (d, *J =* 8.0 Hz, 1H), δ 7.41 (t, *J =* 8.4 Hz, 1H), δ 3.94 (s, 3H). ^13^C APT (DMSO-d_6_; 176 MHz): δ 160.69, 152.52, 138.06, 130.33, 129.98, 127.68, 124.19, 121.10, 117.13, 116.54, 52.99. ESI-MS (m/z): 254.0 [M + H]^+^, 267.1 [M + Na]^+^, 283.1 [M + K] ^+^.

**Methyl 7-methoxy-4-oxo-4, 5-dihydro-[1, 2, 3]triazolo[1,5-*a*]quinoxaline-3-carboxylate (5d).** White solid, 56% yield. ^1^H NMR (DMSO-d_6_, 400 MHz): δ 12.16 (s, 1H), δ 8.29 (d, *J =* 8.8 Hz, 1H), δ 7.01 (d, *J =* 9.2 Hz, 1H), δ 6.94 (s, 1H), δ 3.93 (s, 3H), δ 3.85 (s, 3H). ^13^C APT (DMSO-d_6_; 176 MHz): δ 160.72, 160.53, 152.71, 137.92, 131.49, 126.62, 117.91, 115.26, 111.41, 100.61, 56.24, 52.94. ESI-MS (m/z): 275.2 [M + H]^+^, 297.2 [M + Na]^+^, 313.3 [M + K] ^+^.

**Methyl 7-fluoro-4-oxo-4, 5-dihydro-[1, 2, 3]triazolo[1,5-*a*]quinoxaline-3-carboxylate (5e).** White solid, 35% yield. ^1^H NMR (DMSO-d_6_, 400 MHz) δ: 12.33 (s, 1H), δ 8.45-8.41 (m, 1H), δ 7.28 (t, *J =* 8.4 Hz, 1H), δ 7.21 (d, *J =* 9.6 Hz, 1H), δ 3.93 (s, 3H). ^13^C APT (DMSO-d_6_; 176 MHz): δ 162.98, 161.59, 160.59, 152.65, 138.08, 131.75, 127.29, 118.90 (118.84) 118.18, 111.63 (111.49), 103.54 (103.38), 53.02. ESI-MS (m/z): 263.1 [M + H]^+^, 285.1 [M + Na]^+^, 301.1 [M + K] ^+^.

**4-Oxo-4, 5-dihydro-[1, 2, 3]triazolo[1,5-*a*]quinoxaline-3-carboxylic acid (6).** Compound **5c** (12 mg, 0.05 mmol) was dissolved in a mixture of tetrahydrofuran and water (0.25M, 3:1) and sodium hydroxide (4 mg, 0.1 mmol) was added. Reaction mixture was stirred at room temperature for 1.5 h. One drop of concentrated HCl was then added and the solvent was evaporated under vacuum and the crude product was triturated using dichloromethane, giving compound **6** as white solid (11 mg, quantitative). ^1^H NMR (DMSO-d_6_, 400 MHz): δ 13.12 (s, 1H), δ 8.45 (d, 1H, *J =* 8.0 Hz), δ 7.68 (t, 1H, *J =* 7.6 Hz), δ 7.61 (d, 1H, *J =* 8.0 Hz), δ 7.53 (t, 1H, *J =* 8.0 Hz). ^13^C APT (DMSO-d_6_; 176 MHz): δ 159.50, 156.03, 138.64, 130.51, 127.21, 125.46, 121.71, 116.47. ESI-MS (m/z): 231.1 [M + H]^+^, 253.1 [M + Na]^+^.

**Methyl 5-methyl-4-oxo-4,5-dihydro-[1, 2, 3]triazolo[1,5-*a*]quinoxaline-3-carboxylate (7).** Methyl 4-oxo-4,5-dihydro-[1,2,3]triazolo [1,5-a]quinoxaline-3-carboxylate (10 mg, 0.04 mmol), dimethyl carbonate (1.6 mmol; 40 equivalents) and potassium carbonate (0.12 mmol; 3 equivalents) were charged in a closed vessel and the reaction was left at 140 °C for 16 h. The mixture was then cooled at room temperature and purified by silica gel chromatography column with dichloromethane/methanol (98:2), to obtain methyl 5-methyl-4-oxo-4,5-dihydro-[1,2,3]triazolo [1,5-a]quinoxaline-3-carboxylate **7** as off white solid (8.3 mg; 78% yield). ^1^H NMR (DMSO-d_6_, 400 MHz): δ 8.49 (d, *J =* 8.0 Hz, 1H), δ 7.76-7.70 (m, 2H), δ 7.51 (t, *J =* 8.0, 1H), δ 3.94 (s, 3H), δ 3.65 (s, 3H). ^13^C APT (DMSO-d_6_; 176 MHz): δ 160.74, 152.20, 138.27, 131.09, 130.59. 126.62, 125.53, 121.55, 117.13, 116.70, 53.06, 29.61. ESI-MS (m/z): 259.1 [M + H]^+^, 281.1 [M + Na]^+^, 297.1 [M + K] ^+^.

**Benzyl 5-benzyl-4-oxo-4,5-dihydro-[1,2,3]triazolo[1,5-a]quinoxaline-3-carboxylate (8).** ^1^H NMR (DMSO-d_6_, 400 MHz): δ 8.51 (d, J = 8.0 Hz, 1 H), δ 7.59-7.24 (m, 13 H), δ 5.52 (s, 2 H), δ 5.46 (s, 2 H). ^13^C APT (DMSO-d_6_; 176 MHz): δ 160.11, 152.71, 138.43, 136.12, 130.44, 130.26, 129.08, 128.95, 128.71, 127.76, 127.11, 126.91, 124.67, 121.96, 117.41, 116.98. 67.35, 45.42. ESI-MS (m/z): 411.2 [M + H]^+^, 433.2 [M + Na]^+^, 449.2 [M + K] ^+^.

**Methyl 5-benzyl-4-oxo-4, 5-dihydro-[1, 2, 3]triazolo[1,5-*a*]quinoxaline-3-carboxylate (9).** To a suspension of methyl 4-oxo-4,5-dihydro-[1,2,3]triazolo [1,5-a]quinoxaline-3-carboxylate (5 mg, 0.02 mmol) in cyclopentyl methyl ether (0.1M) in a closed vessel, benzyl bromide (0.03 mmol, 3.6 μL) and potassium carbonate (0.06 mmol, 8.3 mg) were charged and the reaction was left at 130 °C for 12 h. The mixture was then cooled at room temperature and purified by silica gel chromatography column with dichloromethane/methanol (98:2), to obtain methyl 5-benzyl-4-oxo-4,5-dihydro-[1,2,3]triazolo [1,5-a]quinoxaline-3-carboxylate as light yellow solid (6.6 mg, quantitative) and traces of the di-substituted compound **8**. ^1^H NMR (DMSO-d_6_, 400 MHz): δ 8.51 (d, *J =* 8.4 Hz, 1H), δ 7.59 (t, *J =* 8.0Hz, 1H), δ 7.52-7.46 (m, 2H), δ 7.37-7.24 (m, 5H), δ 5.54 (s, 2H), δ 3.95 (s, 3H). ^13^C APT (DMSO-d_6_; 176 MHz): δ 160.73, 152.66, 138.45, 136.08, 130.45, 130.26, 129.07, 127.75, 127.09, 126.75, 124.68, 121.94, 117.49, 116.98, 53.05, 45.42. ESI-MS (m/z): 335.2 [M + H]^+^, 357.2 [M + Na]^+^, 373.1 [M + K] ^+^.

**Methyl 5-(cyclopropylmethyl)-4-oxo-4,5-dihydro-[1,2,3]triazolo[1,5-*a*]quinoxaline-3-carboxylate (10).** To a suspension of methyl 4-oxo-4,5-dihydro-[1,2,3]triazolo [1,5-*a*]quinoxaline-3-carboxylate (5 mg, 0.02 mmol) in cyclopentyl methyl ether (0.1M) in a round-bottom flask, cyclopropyl methyl bromide (0.03mmol, 3.0 μL) and potassium carbonate (0.06 mmol, 8.3 mg) were added and the reaction was left at reflux for 12 h. The mixture was then cooled at room temperature and purified by silica gel chromatography column with *n*-hexane/ethyl acetate (80:20), to obtain **8** as white solid, 86% yield. ^1^H NMR (DMSO-d_6_, 400 MHz): δ 8.52 (dd, J = 8.0, 1.2 Hz, 1H), δ 7.88 (d, *J =* 8.0 Hz, 1H), δ 7.73 (t, 1H, *J =* 8.4 Hz), δ 7.52 (t, 1H, *J =* 8.0 Hz), δ 4.22 (d, *J =* 6.8 Hz, 2H), δ 3.94 (s, 3H) δ, 1.31-1.26 (m, 1H), δ 0.51-0.49 (m, 4H). ^13^C APT (DMSO-d_6_; 176 MHz): δ 160.71, 152,34, 138,37, 130.65, 130.34, 126.47, 124.56, 121.64, 117.36, 117.03, 53.05, 45.94, 10.02, 4.37. ESI-MS (m/z): 299.2 [M + H]^+^, 321.2 [M + Na]^+^, 337.2 [M + K] ^+^.

#### 3.1.6 General procedures for coupling reactions

##### 3.1.6.1 **Method A** (for primary, secondary and benzylamines)

To Methyl 4-oxo-4,5-dihydro-[1, 2, 3]triazolo [1,5-*a*] quinoxaline-3-carboxylate or Methyl 5-methyl-4-oxo-4,5-dihydro-[1, 2, 3]triazolo [1,5-*a*] quinoxaline-3-carboxylate or Methyl 5-benzyl-4-oxo-4,5-dihydro-[1, 2, 3]triazolo [1,5*-a*] quinoxaline-3-carboxylate, the corresponding amines were added (benzylamine, 3-picolylamine, cyclohexylamine, pyrrolidine). Reaction was performed under ultrasound bath at room temperature for 15 min. The mixture was then purified by silica gel chromatography column with dichloromethane/methanol (98:2), to obtain the corresponding amides.

***N*-benzyl-4-oxo-4, 5-dihydro-[1,2,3]triazolo[1,5-*a*]quinoxaline-3-carboxamide (11).** White solid, quantitative yield. ^1^H NMR (DMSO-d_6_, 400 MHz): δ 10.74 (s, 1H), δ 8.42 (d, *J =* 8.4 Hz, 1H), δ 7.62 (t, *J =* 8.4 Hz, 1H), δ 7.52-7.34 (m, 6H), δ 7.28 (t, *J =* 6.8 Hz, 1H), δ 4.62 (d, *J =* 5.6 Hz, 2H). ^13^C APT (DMSO-d_6_; 176 MHz): δ 158.64, 156.10, 140.90, 139.18, 130.16, 128.92, 127.80, 127.50, 125.68, 124.72, 121.64, 118.10, 116.42, 42.90. ESI-MS (m/z): 320.2 [M + H]^+^, 342.2 [M + Na]^+^, 358.2 [M + K] ^+^.

***N*-cyclohexyl-4-oxo-4, 5-dihydro-[1,2,3]triazolo[1,5-*a*]quinoxaline-3-carboxamide (12).** White solid, 97% yield. ^1^H NMR (DMSO-d_6_, 400 MHz): δ 10.24 (d, *J =* 7.2 Hz, 1H), δ 8.41 (d, *J =* 8.0 Hz, 1H), δ 7.62 (t, *J =* 8.0 Hz, 1H), δ 7.53-7.45 (m, 2H), δ 3.92-3.85 (m, 1H), δ 1.91-1.85 (m, 2H), δ 1.78–1.72 (m, 2H), δ 1.59-1.53 (m, 1H), δ 1.44-1.29 (m, 5H). ^13^C APT (DMSO-d_6_; 176 MHz): δ 157.44, 156.04, 141.37, 130.19, 125.39, 124.89, 121.63, 117.79, 116.47, 47.81, 32.51, 25.69, 24.29. ESI-MS (m/z): 312.3 [M + H]^+^, 334.2 [M + Na]^+^, 350.2 [M + K] ^+^.

**3-(Pyrrolidine-1-carbonyl)-[1,2,3]triazolo[1,5-*a*]quinoxalin-4(5*H*)-one (13).** White solid, 99% yield. ^1^H NMR (DMSO-d_6_, 400 MHz): δ 12.16 (s, 1H), δ 7.36 (d, *J =* 8.4 Hz, 1H), δ 7.59 (t, *J =* 8.0 Hz, 1H), δ 7.48-7.40 (m, 2H), δ 3.56-3.51 (m, 2H), δ 3.30-3.28 (m, 2H), δ 1.95-1.88 (m, 2H), δ 1.86-1.79 (m, 2H). ^13^C APT (DMSO-d_6_; 176 MHz): δ 159.53, 153.57, 142.52, 130.07, 129.89, 124.59, 124.25, 121.31, 117.39, 116.38, 47.84, 46.02, 25.81, 24.45. ESI-MS (m/z): 284.2 [M + H]^+^.

***N*-benzyl-5-methyl-4-oxo-4,5-dihydro-[1,2,3]triazolo[1,5-*a*]quinoxaline-3-carboxamide (14).** White solid, 94% yield. ^1^H NMR (DMSO-d_6_, 400 MHz): δ 10.48 (t, *J =* 5.6 Hz, NH, 1H), δ 8.53 (d, *J =* 8 Hz, 1H), δ 7.81 (d, *J =* 8.4 Hz, 1H), δ 7.75 (t, *J =* 7.6 Hz, 1H), δ 7.57 (t, *J =* 8.0 Hz, 1H), δ 7.42-7.35 (m, 4H), δ 7.29 (t, *J =* 7.2 Hz, 1H), δ 4.63 (d, *J =* 6.0 Hz, 2H), δ 3.69 (s, 3H). ^13^C APT (DMSO-d_6_; 176 MHz): δ 158.48, 155.28, 141.23, 139.15, 130.51, 130.44, 128.98, 127.89, 127.57, 125.35, 124.82, 122.00, 117.53, 116.68, 42.95, 30.12. ESI-MS (m/z): 334.2 [M + H]^+^.

**5-Methyl-4-oxo-*N*-(pyridin-3-yl methyl)-4, 5-dihydro-[1, 2, 3]triazolo[1,5-*a*]quinoxaline-3-carboxamide (15).** White solid, 95% yield. ^1^H NMR (DMSO-d_6_, 400 MHz): δ 10.49 (t, *J =* 5.2 Hz, 1H), δ 8.64 (s, 1H), δ 8.54-8.49 (m, 2H), δ 7.81 (d, *J =* 8.4 Hz, 2H), δ 7.75 (t, *J =* 7.6, 1H), δ 7.58 (t, *J =* 7.6 Hz, 1H), δ 7.42-7.38 (m, 1H), δ 4.66 (d, *J =* 6.0 Hz, 2H), δ 3.70 (s, 3H). ^13^C APT (DMSO-d_6_; 176 MHz): δ 158.72, 155.20, 149.33, 148.85, 141.09, 135.76, 134.81, 130.54, 130.45, 125.36, 124.91, 124.09, 121.97, 117.54, 116.68, 40.63, 30.12. ESI-MS (m/z): 335.2 [M + H]^+^.

**5-Benzyl-4-oxo-*N*-(pyridin-3-yl methyl)-4, 5-dihydro-[1, 2, 3]triazolo[1,5-*a*]quinoxaline-3-carboxamide (16).** White solid, 96% yield. ^1^H NMR (DMSO-d_6_, 400 MHz): δ 10.39 (t, *J =* 6.0Hz, 1H), δ 8.62 (s, 1H), δ 8.55 (d, *J =* 8.0 Hz, 1H), δ 8.47 (d, *J =* 4.4 Hz, 1H), δ 7.81 (d, *J =* 7.6 Hz, 1H), δ 7.63-7.51 (m, 3H), δ 7.39-7.25 (m, 6H), δ 5.59 (s, 2H), δ 4.65 (d, *J =* 5.8 Hz, 2H). ^13^C APT (DMSO-d_6_; 176 MHz): δ 158.85, 156.65, 149.31, 148.80, 141.39, 135.81, 135.70, 134.85, 130.39, 129.63, 129.12, 127.86, 127.04, 125.46, 124.09, 122.34,117.78, 116.94, 45.87, 40.64. ESI-MS (m/z): 411.2 [M + H]^+^

***N*-5-Dibenzyl-4-oxo-4, 5-dihydro-[1,2,3]triazolo[1,5*-a*]quinoxaline-3-carboxamide (17).** White solid, quantitative. ^1^H NMR (DMSO-d_6_, 400 MHz): δ 10.38 (t, *J =* 5.6 Hz, 1H, NH), δ 8.55 (d, *J =* 7.6 Hz, 1H), δ 7.63-7.51 (m, 3H), δ 7.41-7.25 (m, 10H), δ 5.58 (s, 2H), δ 6.62 (d, *J =* 5.6 Hz, 2H). ^13^C APT (DMSO-d_6_; 176 MHz): δ 158.60, 155.73, 141.52, 139.15, 135.70, 130.37, 129.62, 129,12, 128.96, 127.92, 127.85, 127.57, 127.03, 125.45, 124.97, 122.37, 117.78, 116.94, 45.87, 42.96. ESI-MS (m/z): 410.3 [M + H]^+^.

**5-Benzyl-4-oxo-*N*-(3-(trifluoromethyl)benzyl**)**-4, 5-dihydro-[1,2,3]triazolo[1,5-*a*]quinoxaline-3-carboxamide (18).** White solid, 89% yield. ^1^H NMR (DMSO-d_6_, 400 MHz): δ 10.42 (t, *J =* 6.0 Hz, 1H), δ 8.55 (d, *J =* 8.0 Hz, 1H), δ 7.78 (s, 1H), δ 7.72 (d, *J =* 7.6 Hz, 1H), δ 7.65-7.51 (m, 5H), δ 7.39 (d, *J =* 7.2 Hz, 2H), δ 7.34-7.25 (m, 3H), δ 5.59 (s, 2H), δ 4.72 (d, *J =* 6.0 Hz, 2H). ^13^C APT (DMSO-d_6_; 176 MHz): δ 158.90, 155.65, 141.42, 140.93, 135.78, 132.05, 130.37, 129.99, 129.67, 129.08, 127.85, 127.14, 125.41, 125.13, 124.38, 124.26, 122.38, 117.78, 116.94, 45.85, 42.47. ESI-MS (m/z): 478.3 [M + H]^+^.

**5-Benzyl-*N*-(4-chlorobenzyl)-4-oxo-4, 5-dihydro-[1, 2, 3]triazolo[1,5-*a*]quinoxaline-3-carboxamide (19).** White solid, 93% yield. ^1^H NMR (DMSO-d_6_, 400 MHz): δ 10.37 (t, *J =* 6.0 Hz, 1H, NH), δ 8.55 (d, *J =* 8.0 Hz, 1H), δ 7.64-7.51 (m, 3H), δ 7.42 (s, 3H), δ 7.39-7.25 (m, 6H), δ 5.59 (s, 2H), δ 4.62 (d, *J =* 6.0 Hz, 2H). ^13^C APT (DMSO-d_6_; 176 MHz): δ 158.68, 155.70, 141.45, 138.37, 135.75, 132.10, 130.36, 129.82, 129.66, 129.10, 128.88, 127.83, 127.07, 125.42, 125.05, 122.39, 117.81, 116.93, 45.87, 42.27. ESI-MS (m/z): 444.3 [M + H]^+^.

##### 3.1.6.2 **Method B** (for anilines)

**4-Oxo-*N*-phenyl-4, 5-dihydro-[1,2,3]triazolo[1,5-*a*]quinoxaline-3-carboxamide (20).** To a solution of Methyl 4-oxo-4,5-dihydro-[1,2,3]triazolo [1,5-a]quinoxaline-3-carboxylate (7.3 mg; 0.03 mmol) in cyclopentyl methyl ether (0.3 M), aniline (0.039 mmol, 3.55 μL) and Cp_2_ZrCl_2_ (10 mol%, 0.003 mmol, 0.9 mg) were added. The mixture was refluxed for 12 h, then cooled and purified by silica gel chromatography column with dichloromethane/methanol (98:2), to obtain compound **20** as white solid, 81% yield. ^1^H NMR (DMSO-d_6_, 400 MHz): δ 12.45 (s, 1H), δ 8.46 (d, *J =* 8.0 Hz, 1H), δ 7.76 (d, *J =* 8.0 Hz, 2H), δ 7.67 (t, *J =* 7.6 Hz, 1H), δ 7.57 (d, *J =* 8.0 Hz, 1H), δ 7.51 (t, *J =* 7.6 Hz, 1H), δ 7.44 (t, *J =* 7.6 Hz, 2H), δ 7.17 (t, *J =* 7.2 Hz, 1H). ^13^C APT (DMSO-d_6_; 176 MHz): δ 156.60, 156.37, 141.30, 138.93, 130.38, 129.69, 125.65, 125.13, 124.59, 121.67, 119.84, 116.53. ESI-MS (m/z): 306.2 [M + H]^+^

### 3.2 Biological procedures

#### 3.2.1 Antibiotics and strains

Vancomycin (VAN), oxacillin (OXA), imipenem (IPM), tobramycin (TOB), amphotericin B (AMB), and voriconazole (VRC) were purchased from Sigma-Aldrich (Milan, Italy). *Staphylococcus aureus* ATCC 29213, ATCC 43300 (methicillin-resistant *S. aureus*, MRSA), *S. epidermidis* ATCC 35984, *Pseudomonas aeruginosa* ATCC 27853, *Klebsiella pneumoniae* ATCC 13883, *K. pneumonia* ATCC BAA-1705 (carbapenem-resistant) *Candida albicans* ATCC 90028, and *Candida albicans* ATCC 10231 (voriconazole resistant strain) were obtained from the American Type Culture Collection (Rockville, MD).

#### 3.2.2 Antimicrobial susceptibility testing

Minimal inhibitory concentrations (MIC) of all the compounds were determined in Mueller–Hinton medium (MH) by the broth microdilution assay, following the procedure already described (Fiorani et al., 2018). The compounds were added to the bacterial suspension in each well yielding a final cell concentration of 1 × 10^6^ CFU/ml and a final compound concentration ranging from 3,1–100 μM. Negative control wells were set to contain bacteria in Mueller–Hinton broth plus the amount of vehicle (DMSO) used to dilute each compound. Positive controls included VAN (2 μg/ml), OXA (2 μg/ml), IPM (2 μg/ml), and TOB (2 μg/ml), the MIC was defined as the lowest concentration of drug that caused a total inhibition of microbial growth after 24 h incubation time at 37°C

The antifungal activity of compounds was determined using a standardized broth microdilution method (Clinical and Laboratory Standards Institute. Reference method for broth dilution antifungal susceptibility testing of yeasts. M27—4th Ed. Pennsylvania (US): Clinical and Laboratory Standards Institute; 2017.) Briefly, the cell suspension was adjusted to 3 × 10^3^ CFU/ml in RPMI 1640 medium (Sigma) supplemented with 0.2% (w/v) glucose. One hundred microliter aliquots of these cell suspensions were dispensed into 96-well microtiter plates. Compounds were serially diluted using RPMI 1640 medium and added to the wells at a final concentration ranging from 0.4 to 100 μM, and the plate was incubated for 48 h at 37°C. Voriconazole (30 μg/ml for ATCC 10231 and 0.25 μg/ml for ATCC 90028) and AMB (0.12 μg/ml) were chosen as positive controls.

All the tests were conducted at least three times using independent cell suspensions.

#### 3.2.3 Biofilm formation assay

Biofilm formation was evaluated by measuring the ability of cells to adhere to a sterile 96-well polystyrene flat-bottom microtiter plate (BD Falcon, Mississauga, Ontario Canada) as described previously 18. Briefly, a suspension of *S. epidermidis* (MH supplemented with 1% glucose) at the final density of 10^5^ CFU ml-1 was treated with compounds **9**, **14**, and **20** 50 mM. After 24 h at 37°C, planktonic cells were removed, and the wells washed twice with phosphate-buffered saline (PBS) and dried at 60°C for 30 min. Crystal violet solution (150 ml at 0.1%) was added to each well and the plates were incubated at room temperature for 30 min. The wells were then washed with PBS and discolored with 200 ml of 96% ethanol for 20 min. Absorbance was measured at 620 nm using a microtiter plate reader. The percentage of biofilm mass reduction was calculated using the formula [(Ac-At)/Ac] ×100, where Ac is the absorbance value (OD) for control wells and At is the OD value in the presence of a compound.

#### 3.2.4 Statistical analysis

Statistical analyses for biological assays were performed using GraphPad Prism 9 (GraphPad Software, San Diego, CA, USA). Analysis of variance (ANOVA) for multiple comparisons followed by Dunnett’s *post hoc* test was used to compare the treated and control groups. *p-value* < 0.01 was considered significant for all the *in vitro* experiments.

## 4 Conclusion

We reported the discovery of a novel eco-sustainable protocol for both the synthesis and decoration of the [1,2,3]-triazolo [1,5-*a*]quinoxalin-4(5*H*)-one scaffold, a so far poorly explored moiety for medicinal chemistry purposes. Our conceived procedure rejuvenated the chemical path toward this scaffold by using eco-sustainable reagents, catalysts and neat conditions for the majority of the required synthetic steps. Notably, in the case of aliphatic amines and benzylamines the amidation products were obtained under neat conditions. Furthermore, catalytic direct amidation with anilines was explored for the first time on this scaffold, using dichlorobis (cyclopentadienyl)zirconium (Cp_2_ZrCl_2_) and CPME as eco-friendly catalyst and solvent, respectively.

The results of the antimicrobial assay obtained against *S. aureus* ATCC 29213 and *S. aureus*, *P. aeruginosa*, *K. pneumoniae*, *C. albicans*, and also the inhibition of *S. epidermidis* biofilm formation foster a further optimization campaign, since three out of 16 compounds, namely compounds **9**, **14** and **20**, displayed a limited antimicrobial property. Interestingly compound **9** reduced the growth of different bacteria and the yeast *Candida*, also affecting biofilm formation in *S. epidermidis*, thus proving to be a promising hit compound for the discovery of novel agents against polymicrobial infections.

## Data Availability

The original contributions presented in the study are included in the article/Supplementary Material, further inquiries can be directed to the corresponding authors.

## References

[B1] Ager I. R. Barnes A. C. Danswan G. W. Hairsine P. W. Kay D. P. Kennewell P. D. (1988). Synthesis and oral antiallergic activity of carboxylic acids derived from imidazo[2,1-c] [1,4]benzoxazines, imidazo[1,2-a]quinolines, imidazo[1,2-a]quinoxalines, imidazo[1,2-a]quinoxalinones, pyrrolo[1,2-a]quinoxalinones, pyrrolo[2,3-a]quinoxalinones, and imidazo[2,1-b]benzothiazoles. J. Med. Chem. 31 (6), 1098–1115. 10.1021/jm00401a009 2897466

[B2] Alfano A. I. Buommino E. Ferraro M. G. Irace C. Zampella A. Lange H. (2021). Coupling interrupted fischer and multicomponent joullié-ugi to chase chemical diversity: From batch to sustainable flow synthesis of peptidomimetics. ChemMedChem 16 (24), 3795–3809. 10.1002/cmdc.202100474 34585536 PMC9297956

[B3] Alswah M. Bayoumi A. H. Kamal Elgamal K. Elmorsy A. Saleh Ihmaid S. Ahmed H. E. A. (2018). Design, synthesis and cytotoxic evaluation of novel chalcone derivatives bearing triazolo[4,3-a]- quinoxaline moieties as potent anticancer agents with dual EGFR kinase and tubulin polymerization inhibitory effects. Molecules 23, 48. 10.3390/molecules23010048 PMC5943945 29280968

[B4] Amer A. Ayoup M. S. Khattab S. N. Hassan S. Y. Langer V. Senior S. (2010). A regio- and stereo-controlled approach to triazoloquinoxalinyl C-nucleosides. Carbohydr. Res. 345, 2474–2484. 10.1016/j.carres.2010.08.010 20934686

[B5] Ayoup M. S. Ahmed H. E. A. El Massry A. M. Senior S. Khattab S. N. Hassan S. Y. (2016). Synthesis, docking, and evaluation of antimicrobial activity of a new series of acyclo C-nucleosides of 1, 2, 4-triazolo[4, 3-a]quinoxaline derivatives. J. Heterocycl. Chem. 53, 153–163. 10.1002/jhet.2396

[B6] Ayoup M. S. Rabee A. R. Abdel-Hamid H. Harras M. F. El Menofy N. G. Ismail M. M. F. (2022). Exploration of nitroaromatic antibiotics via sanger’s reagent: Synthesis, in silico, and antimicrobial evaluation. ACS Omega 7 (6), 5254–5263. 10.1021/acsomega.1c06383 35187340 PMC8851660

[B7] Baashen M. A. Abdel-Wahab B. F. El-Hiti G. A. (2016). Syntheses of triazoloquinoxalines. Heterocycles 92, 1931–1952. 10.3987/rev-16-847

[B8] Barral K. Moorhouse D. Moses J. E. (2007). Efficient conversion of aromatic amines into Azides: A one-pot synthesis of triazole linkages. Org. Lett. 9, 1809–1811. 10.1021/ol070527h 17391043

[B9] Biagi G. Giorgi I. Livi O. Scartoni V. Betti L. Giannaccini G. (2002). New 1,2,3-triazolo[1,5-a]quinoxalines: Synthesis and binding to benzodiazepine and adenosine receptors. II. II Eur. J. Med. Chem. 37, 565–571. 10.1016/s0223-5234(02)01376-4 12126775

[B10] El-Attar M. A. Z. Elbayaa R. Y. Shaaban O. G. Habib N. S. Abdel Wahab A. E. Abdelwahab I. A. (2018). Synthesis of pyrazolo-1,2,4-triazolo[4,3-a]quinoxalines as antimicrobial agents with potential inhibition of DHPS enzyme. Future Med. Chem. 10, 2155–2175. 10.4155/fmc-2018-0082 30088415

[B11] El-Sagheer A. H. Brown T. (2012). Click nucleic acid ligation: Applications in biology and nanotechnology. Acc. Chem. Res. 45, 1258–1267. 10.1021/ar200321n 22439702 PMC3423825

[B12] Fiorani G. Perosa A. Selva M. (2018). Dimethyl carbonate: A versatile reagent for a sustainable valorization of renewables. Green Chem. 20, 288–322. 10.1039/c7gc02118f

[B13] HongShen C. H. C. Ding F. X. Deng Q. Wilsie L. C. Krsmanovic M. L. Taggart A. K. (2009). Discovery of novel tricyclic full agonists for the G-protein-coupled niacin receptor 109A with minimized flushing in rats. J. Med. Chem. 52, 2587–2602. 10.1021/jm900151e 19309152

[B14] Houa H. Yangb R. Liub X. Wub X. Zhangb S. Chenb K. (2020). Discovery of triazoloquinoxaline as novel STING agonists via structure-based virtual screening. Bioorg. Chem. 100, 103958. 10.1016/j.bioorg.2020.103958 32470762

[B15] Mirzaei H. Eshghi H. Seyedi S. M. (2021). Cu Nano particles immobilized on silk-fibroin as a greenand biodegradable catalyst for copper catalyzed azide-terminal, internal alkynes cycloaddition. Appl. Organomet. Chem. 35, e6019. 10.1002/aoc.6019

[B16] Nagavelli V. R. Nukala S. K. Narsimha S. Battula K. S. Tangeda S. J. Reddy Y. N. (2016). Synthesis, characterization and biological evaluation of 7-substituted-4-((1-aryl-1H-1,2,3-triazol-4-yl) methyl)-2H- benzo[b] [1,4]oxazin-3(4H)-ones as anticancer agents. Med. Chem. Res. 25, 1781–1793. 10.1007/s00044-016-1616-9

[B17] Souad B. Fatmi C. E. Mabrouk T. (2011). Synthesis of some 1,4,5-trisubstituted 1,2,3-triazoles by 1,3-dipolar cycloaddition of 2-substituted phenyl azides to dimethyl acetylene dicarboxylate (DMAD), regular stirring versus microwave irradiation: A comparative study. Rasayan J. Chem. 4, 806–809.

[B18] Vollaro A. Catania M. R. Iesce M. R. Sferruzza R. D’Abrosca B. Donnarumma G. (2019). Antimicrobial and anti-biofilm properties of novel synthetic lignan-like compounds. New Microbiol. 42, 21–28. 30785206

[B19] Watanabe K. Yamagiwa N. Torisawa Y. (2007). Cyclopentyl methyl ether as a new and alternative process solvent. Org. Process Res. Dev. 11 (2), 251–258. 10.1021/op0680136

[B20] Weiss R. Seubert J. Hampel F. (1994). α-Aryliodonio diazo compounds: SN reactions at theα-C atom as a novel reaction Type for diazo compounds. Angew. Chem. Int. Ed. 33 (19), 1952–1953. 10.1002/anie.199419521

[B21] Wen J. Zhao W. Gao X. Ren X. Dong C. Wang C. (2022). Synthesis of [1,2,3]Triazolo-[1,5-a]quinoxalin-4(5H)-ones through photoredox-catalyzed [3 + 2] cyclization reactions with hypervalent iodine(III) reagents. J. Org. Chem. 87, 4415–4423. 10.1021/acs.joc.2c00135 35234036

[B22] Wesseler F. Lohmann S. Riege D. Halver J. Roth A. Pichlo C. (2022). Phenotypic discovery of triazolo[1,5-c]quinazolines as a first-in- class bone morphogenetic protein amplifier chemotype. J. Med. Chem. 65 (22), 15263–15281. 10.1021/acs.jmedchem.2c01199 36346705

[B23] Xu J. Yang H. He L. Huang L. Shen J. Li W. (2021). Synthesis of (E)-quinoxalinone oximes through a multicomponent reaction under mild conditions. Org. Lett. 23, 195–201. 10.1021/acs.orglett.0c03918 33354970

[B24] Yan J. Zhou F. Qin D. Cai T. Ding K. Cai Q. (2012). Synthesis of [1,2,3]Triazolo- [1,5-a]quinoxalin-4(5H)-ones through copper-catalyzed tandem reactions of N-(2-Haloaryl)propiolamides with sodium azide. Org. Lett. 14, 1262–1265. 10.1021/ol300114w 22335274

[B25] Zhang S. Xu Z. Gao C. Ren Q.-C. Chang L. Lv Z.-S. (2017). Triazole derivatives and their anti-tubercular activity. Eur. J. Med. Chem. 138, 501–513. 10.1016/j.ejmech.2017.06.051 28692915

