# Supplementary material for: Rejuvenating the [1, 2, 3]-triazolo [1,5-a]quinoxalin-4(5H)-one scaffold: Synthesis and derivatization in a sustainable guise and preliminary antimicrobial evaluation
Source: Front Chem. 2023 Mar 14;11:1126427. doi: 10.3389/fchem.2023.1126427 (PMC10043301; doi:10.3389/fchem.2023.1126427)
Supplement: Supplementary file 1 [file DataSheet1.PDF]

Supplementary Material

Rejuvenating the [1, 2, 3]-triazolo [1,5-a]quinoxalin-4(5*H*)-one scaffold: synthesis and derivatization in a sustainable guise and preliminary antimicrobial evaluation

Sveva Pelliccia[1,§*], Antonella Ilenia Alfano[1,§], Beatriz Ramos Gomes da Assunção[2], Luigia Turco[1,3], Francesca Lembo[1], Vincenzo Summa[1], Elisabetta Buommino[1], and Margherita Brindisi[1,*]

[1] Department of Pharmacy, University of Naples Federico II, via D. Montesano 49, 80131, Naples, Italy

[2] ESTeSL- Lisbon School of Health Technology, Polytechnic Institute of Lisbon, 1900-096 Lisbon, Portugal

[3] Department of Precision Medicine, University of Campania "Luigi Vanvitelli", 80138 Naples, Italy.

[§] These authors contributed equally to this work

*** Correspondence:**
Corresponding Authors
sveva.pelliccia@unina.it, margherita.brindisi@unina.it

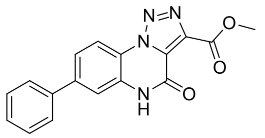

5a

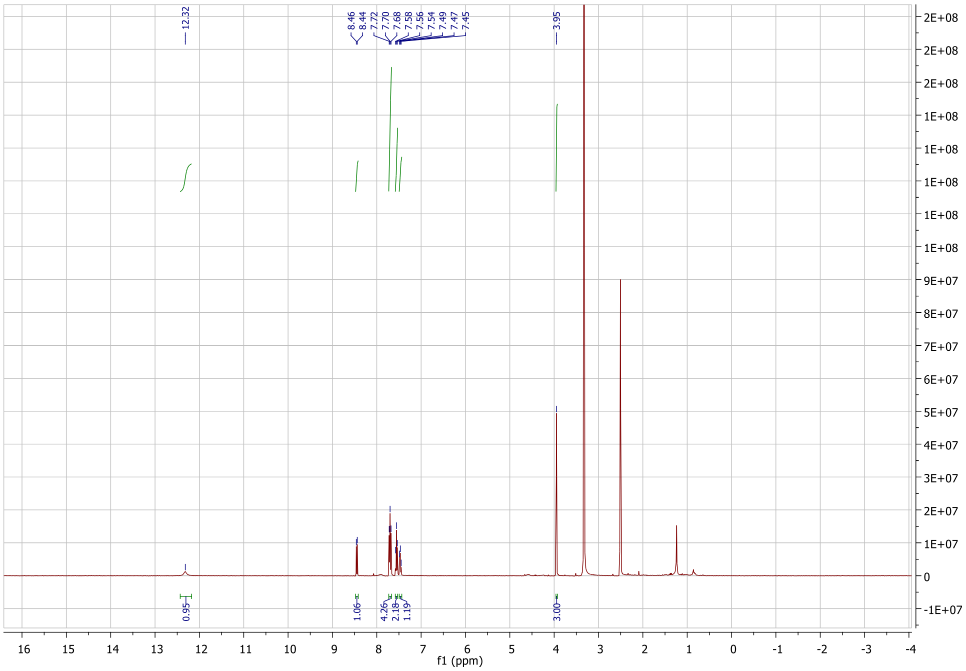
12.32
8.46
8.44
7.72
7.70
7.68
7.58
7.56
7.54
7.49
7.47
7.45
3.95
0.95
1.06
4.26
2.18
1.19
3.00
f1 (ppm)

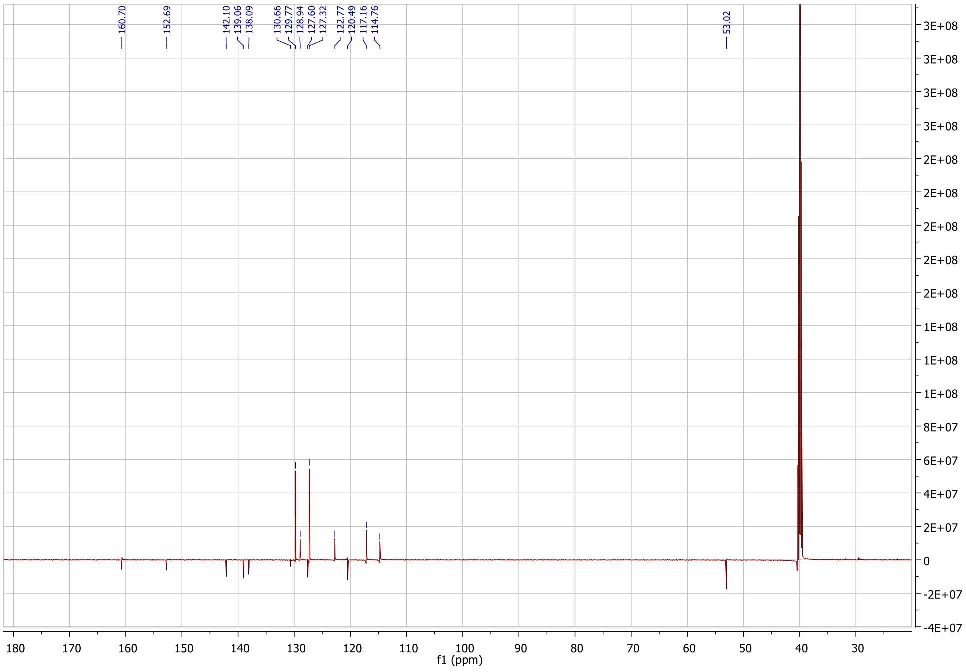
160.70
152.69
142.10
139.06
138.09
130.66
129.77
128.94
127.60
127.32
122.77
120.49
117.16
114.76
53.02
f1 (ppm)

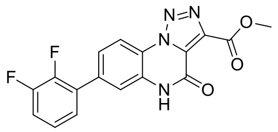

5b

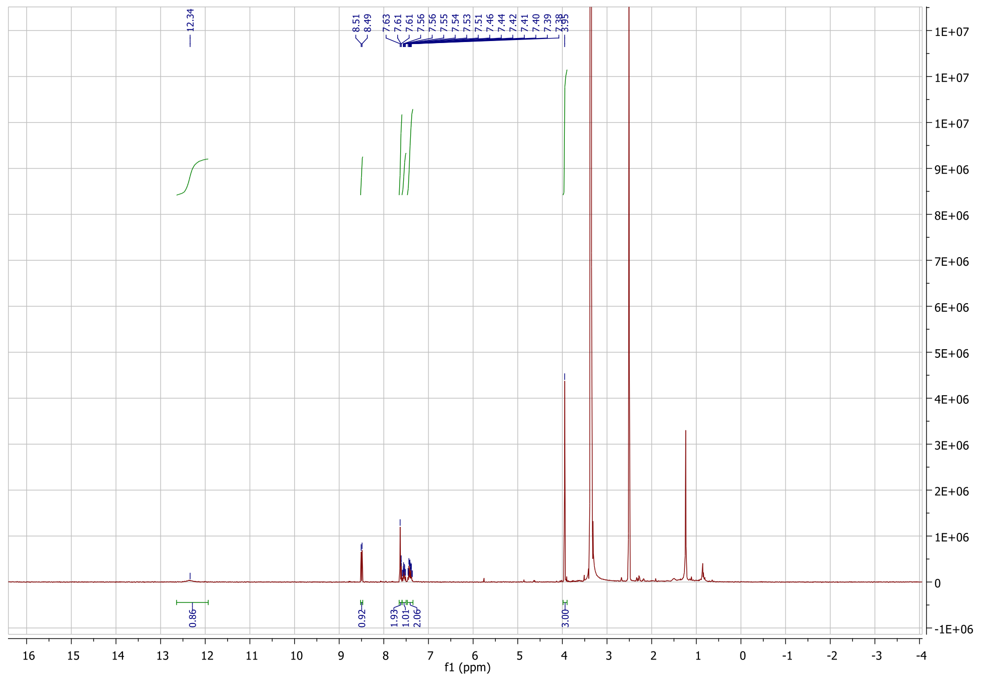
12.34
8.51
8.49
7.63
7.61
7.61
7.56
7.56
7.55
7.54
7.53
7.51
7.46
7.44
7.42
7.41
7.40
7.39
7.38
3.95
0.86
0.92
1.93
1.01
2.06
3.00
f1 (ppm)

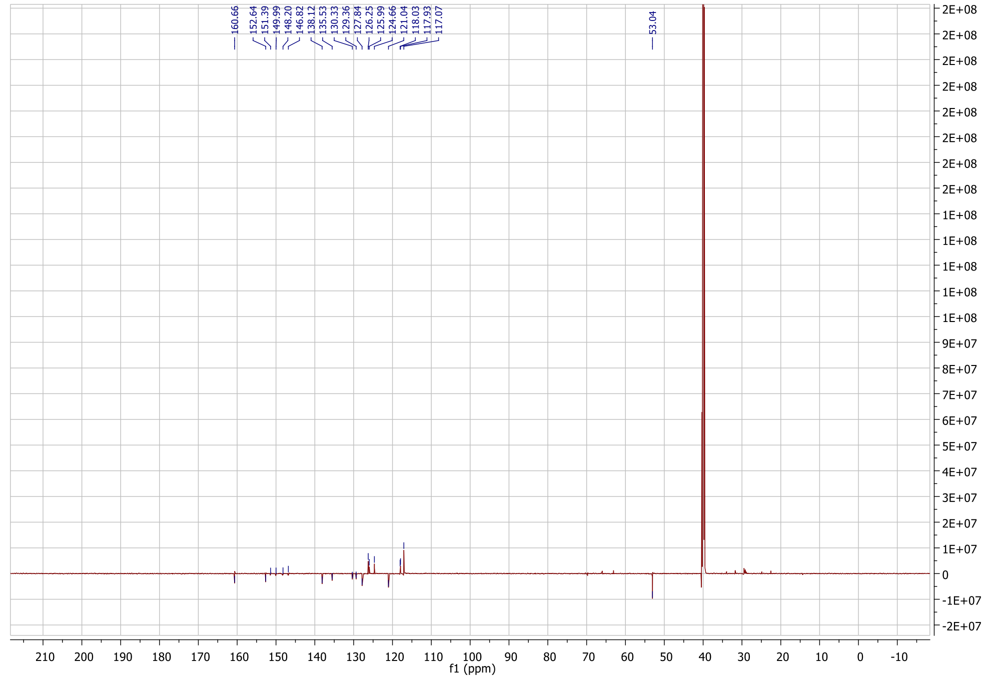
160.66
152.64
151.39
149.99
148.20
146.82
138.12
135.53
130.33
129.36
127.84
126.25
125.99
124.66
121.04
118.03
117.93
117.07
53.04
f1 (ppm)

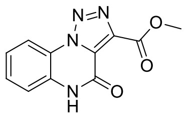

5c

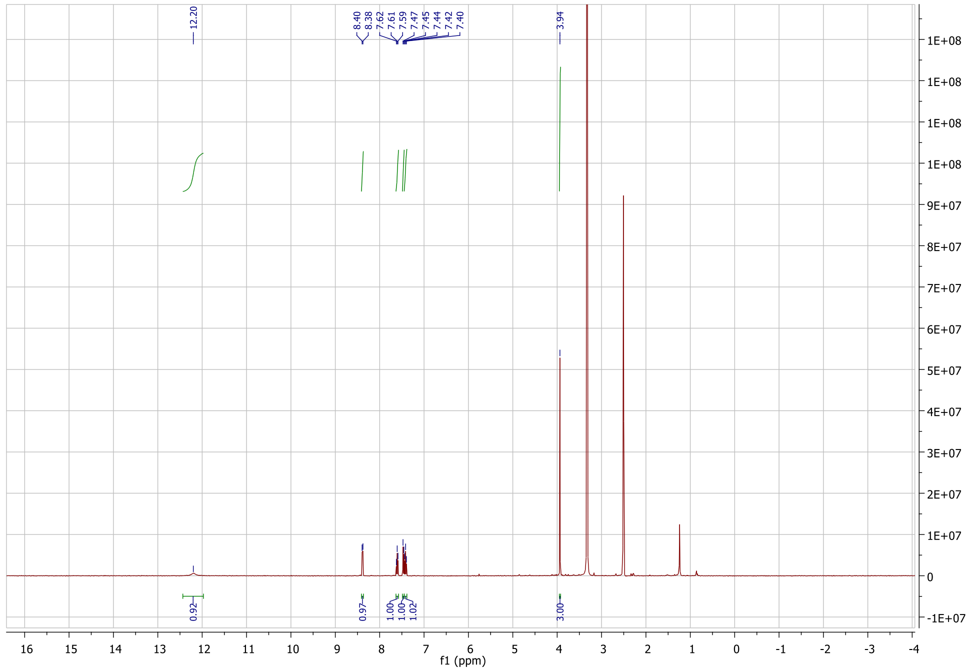
12.20
8.40
8.38
7.62
7.61
7.59
7.47
7.45
7.44
7.42
7.40
3.94
0.92
0.97
1.00
1.00
1.02
3.00
f1 (ppm)

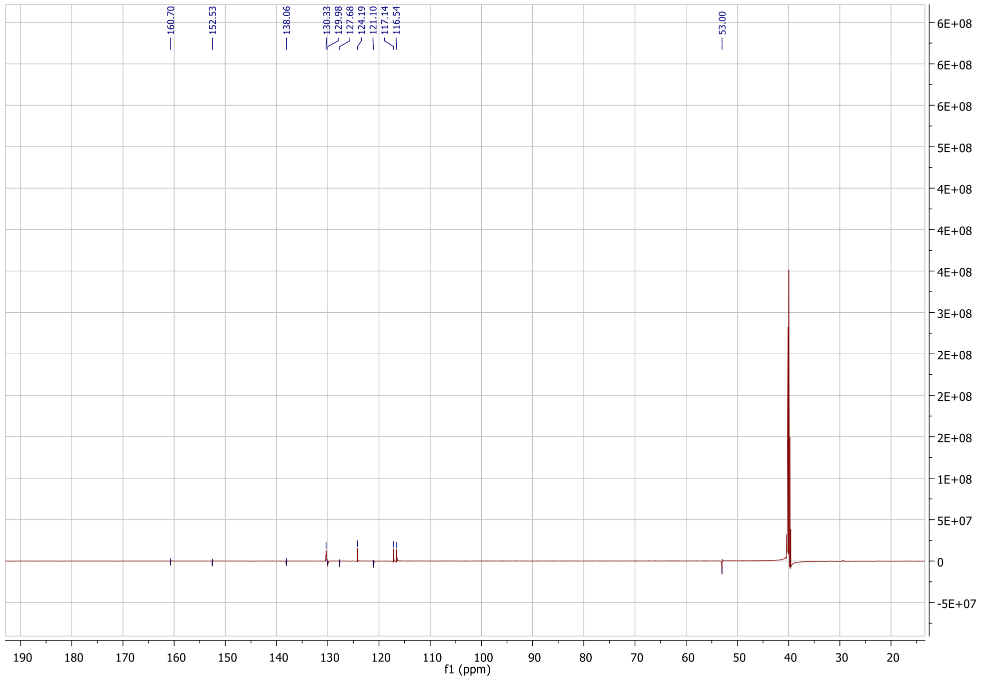
160.70
152.53
138.06
130.33
129.98
127.68
124.19
121.10
117.14
116.54
53.00
f1 (ppm)

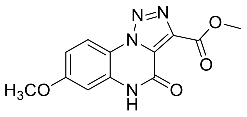

5d

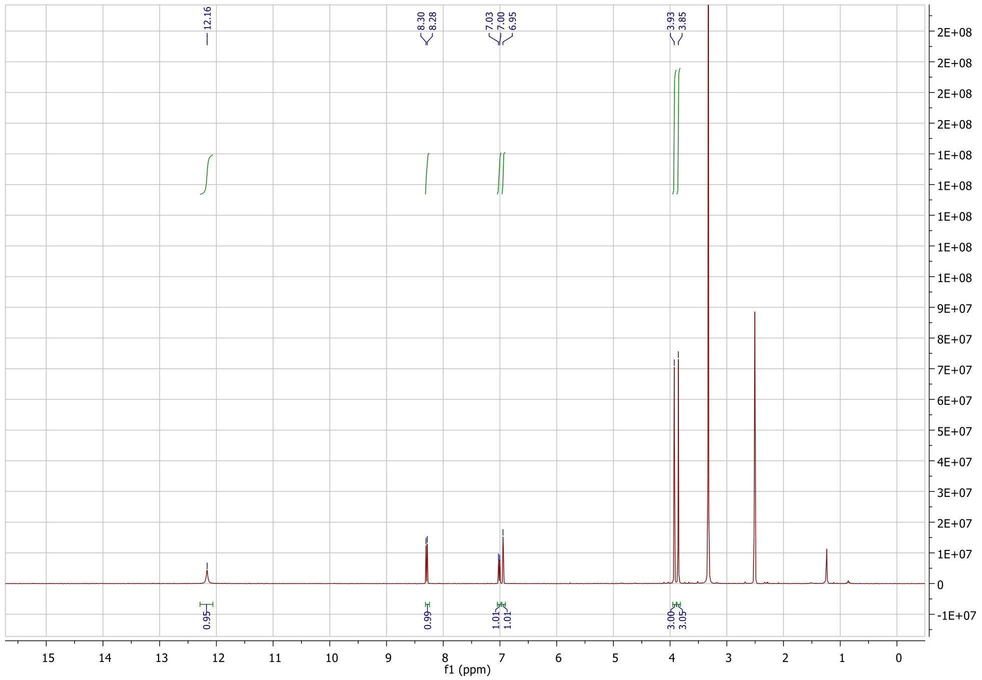
12.16
8.30
8.28
7.03
7.00
6.95
3.93
3.85
0.95
0.99
1.01
1.01
3.00
3.05
f1 (ppm)

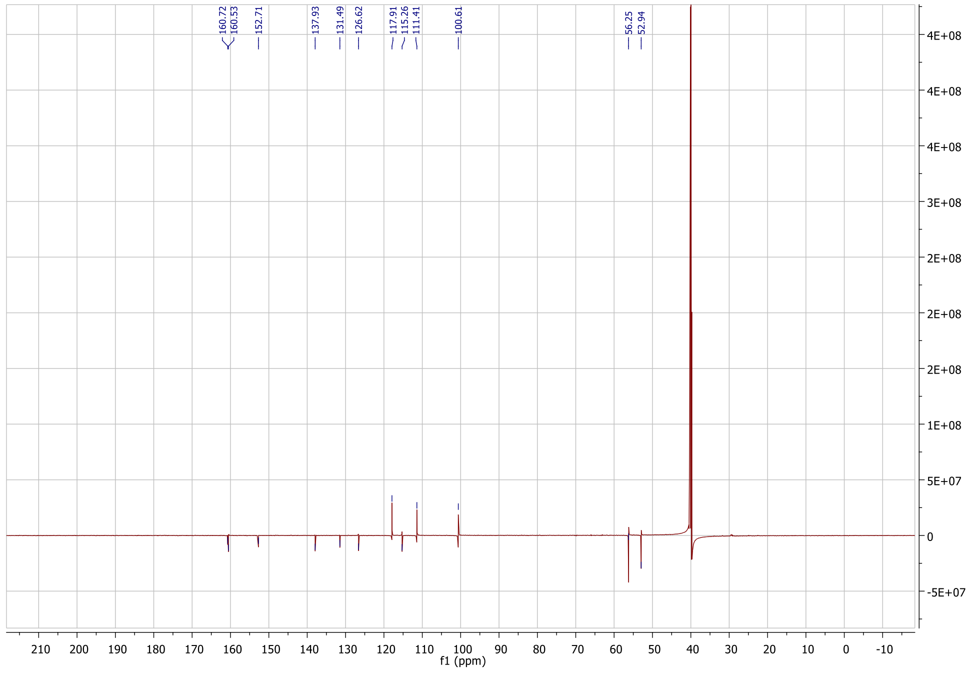
160.72
160.53
152.71
137.93
131.49
126.62
117.91
115.26
111.41
100.61
56.25
52.94
f1 (ppm)

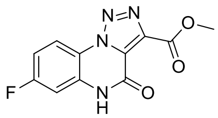

5e

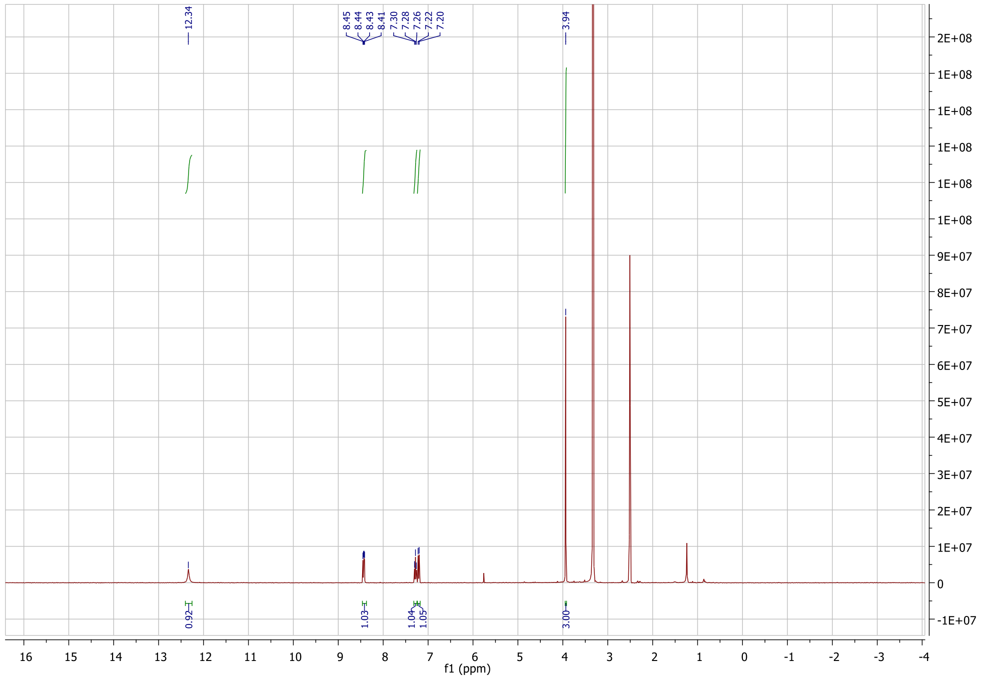
12.34
8.45
8.44
8.43
8.41
7.30
7.28
7.26
7.22
7.20
3.94
0.92
1.03
1.04
1.05
3.00
f1 (ppm)

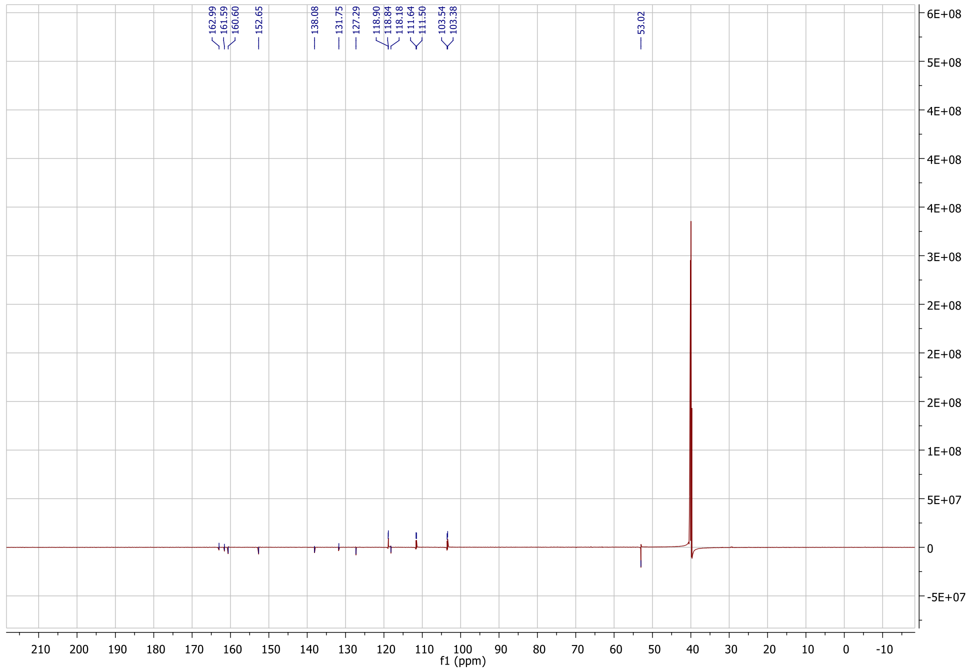
162.99
161.59
160.60
152.65
138.08
131.75
127.29
118.90
118.84
118.18
111.64
111.50
103.54
103.38
53.02
f1 (ppm)

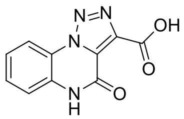
N
N
N
OH
O
O
N
H

6

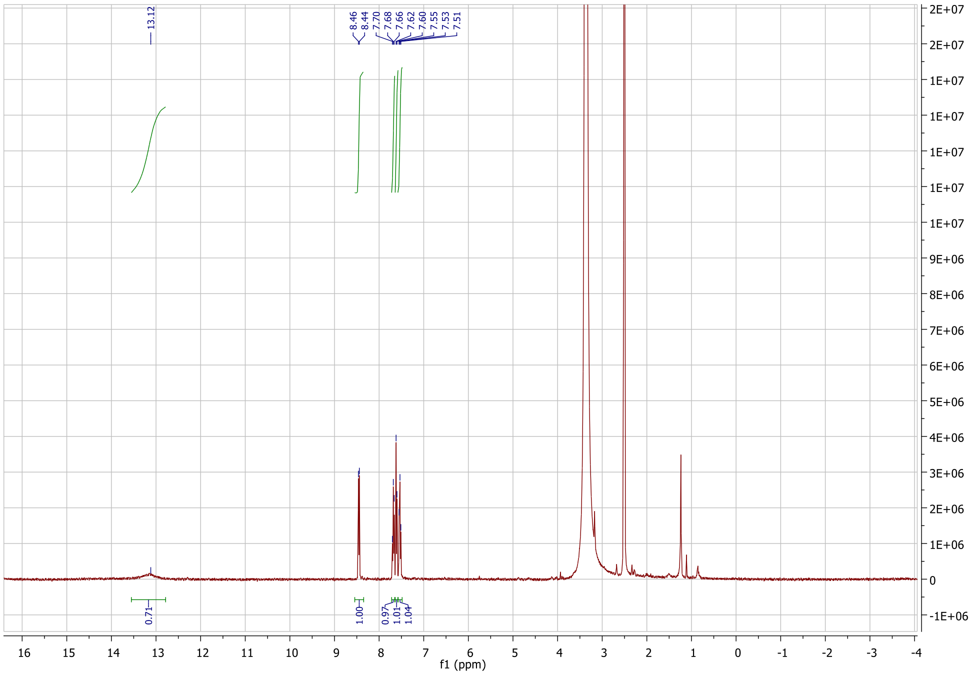
13.12
8.46
8.44
7.70
7.68
7.66
7.62
7.60
7.55
7.53
7.51
0.71
1.00
0.97
1.01
1.04
f1 (ppm)

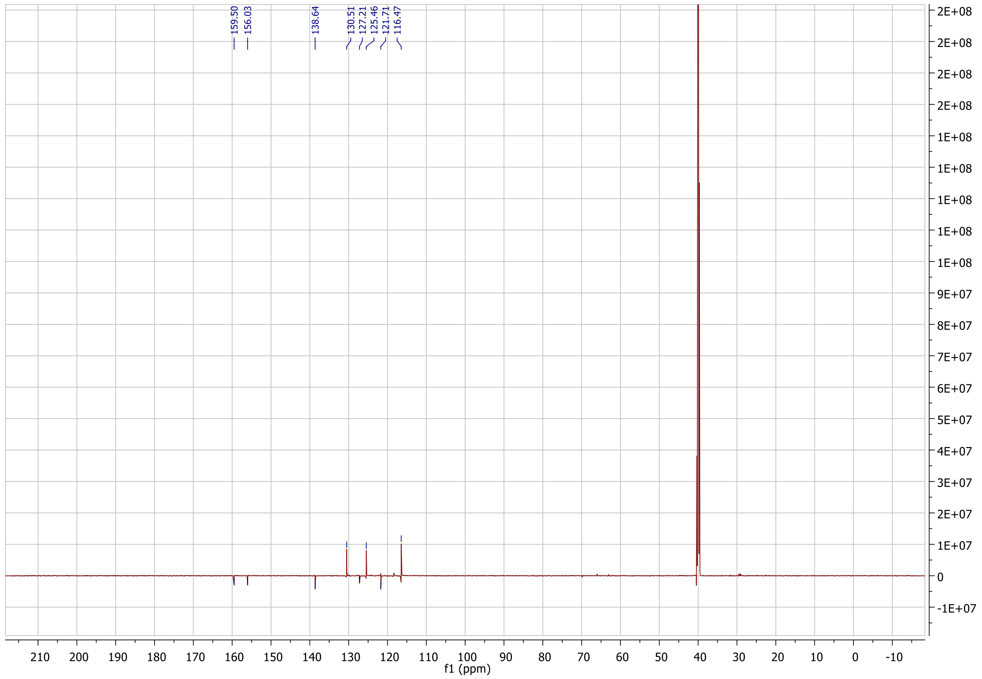
159.50
156.03
138.64
130.51
127.21
125.46
121.71
116.47
f1 (ppm)

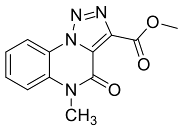
N
N
N
O
O
N
O
CH3

7

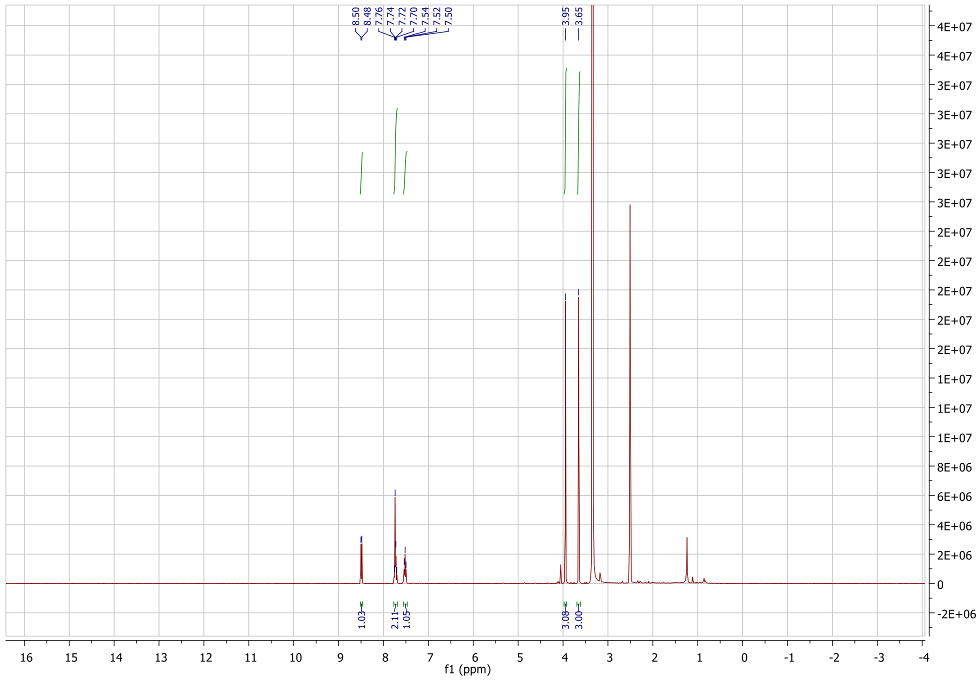
8.50
8.48
7.76
7.74
7.72
7.70
7.54
7.52
7.50
3.95
3.65
1.03
2.11
1.05
3.08
3.00
f1 (ppm)

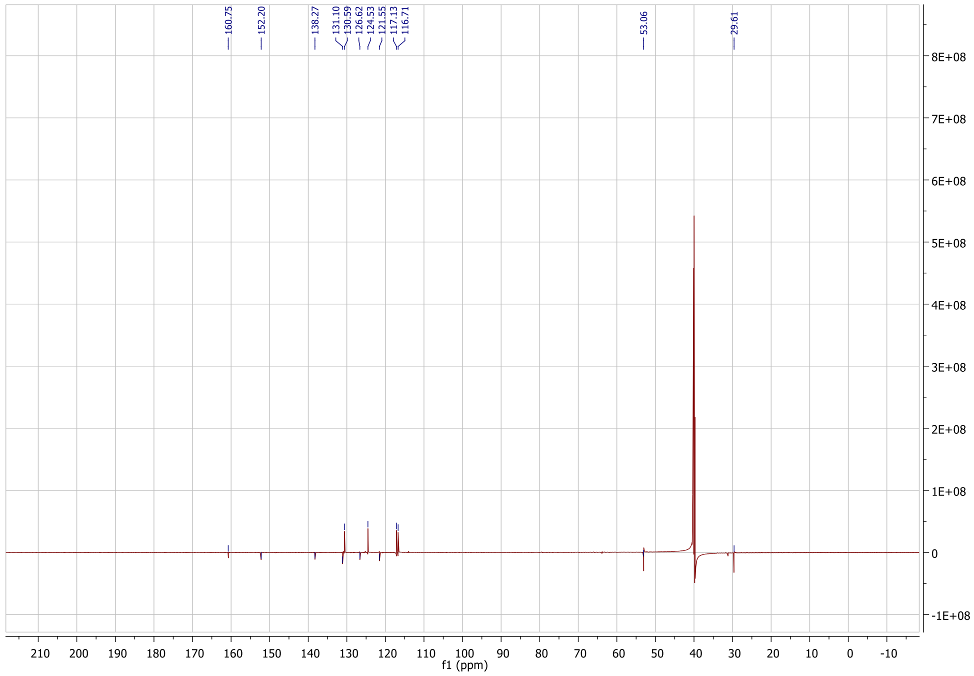
160.75
152.20
138.27
131.10
130.59
126.62
124.53
121.55
117.13
116.71
53.06
29.61
f1 (ppm)

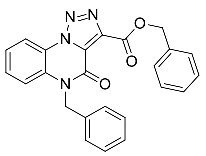

8

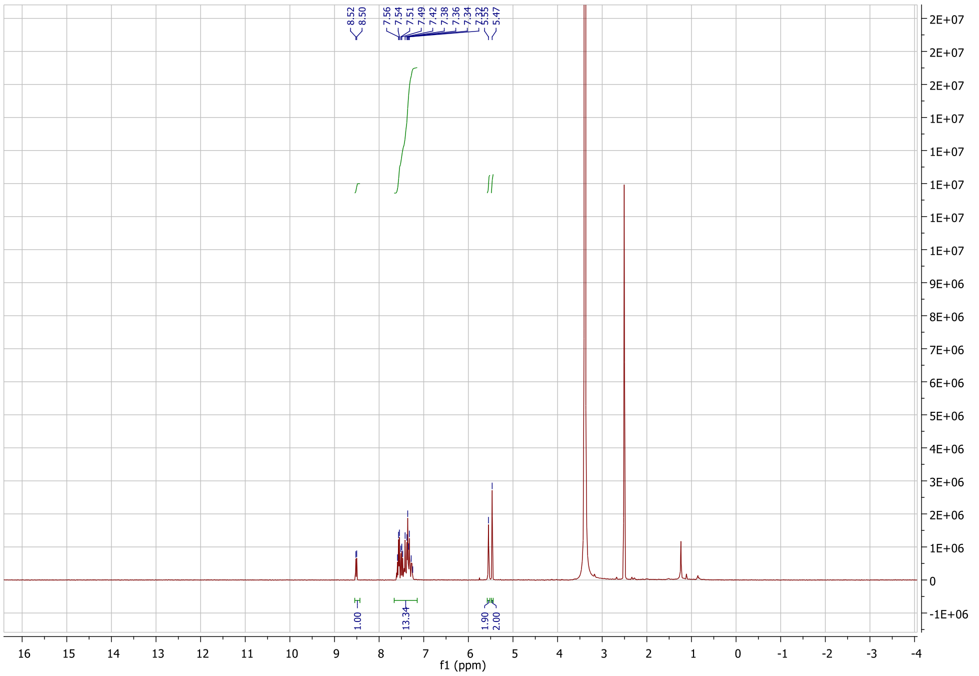
8.52
8.50
7.56
7.54
7.51
7.49
7.42
7.38
7.36
7.34
7.32
5.55
5.47
1.00
13.34
1.90
2.00
f1 (ppm)

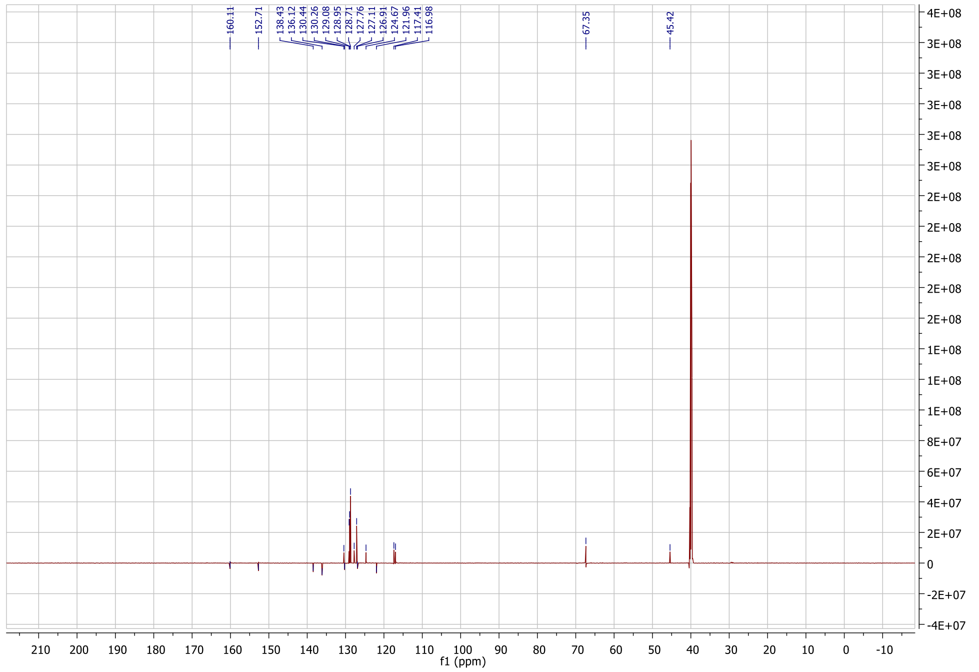
160.11
152.71
138.43
136.12
130.44
130.26
129.08
128.95
128.71
127.76
127.11
126.91
124.67
121.96
117.41
116.98
67.35
45.42
f1 (ppm)

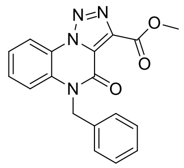

9

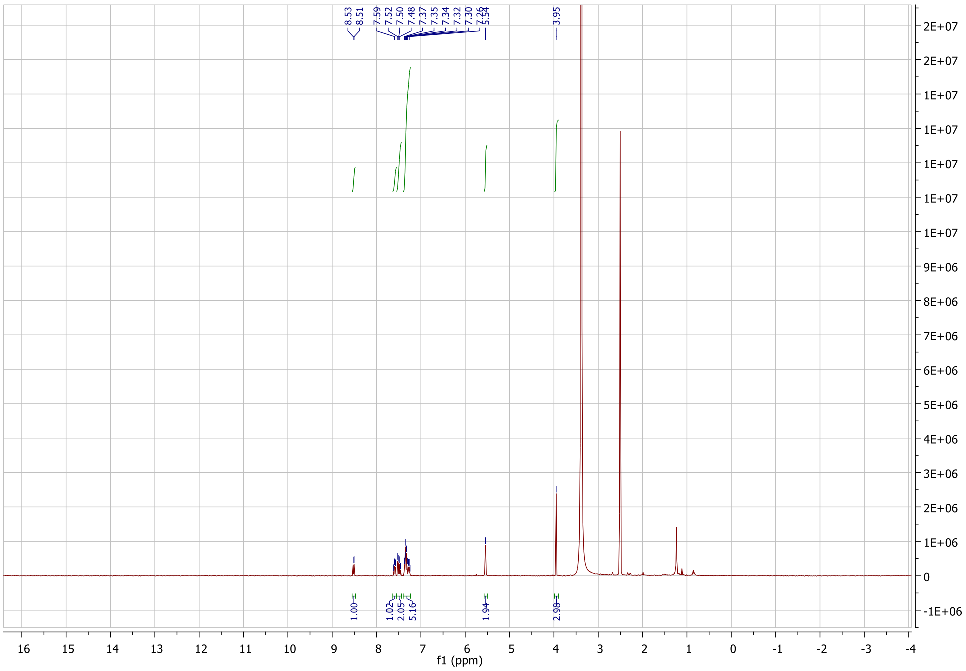
8.53
8.51
7.59
7.52
7.50
7.48
7.37
7.35
7.34
7.32
7.30
7.26
5.54
3.95
1.00
1.02
2.05
5.16
1.94
2.98
f1 (ppm)

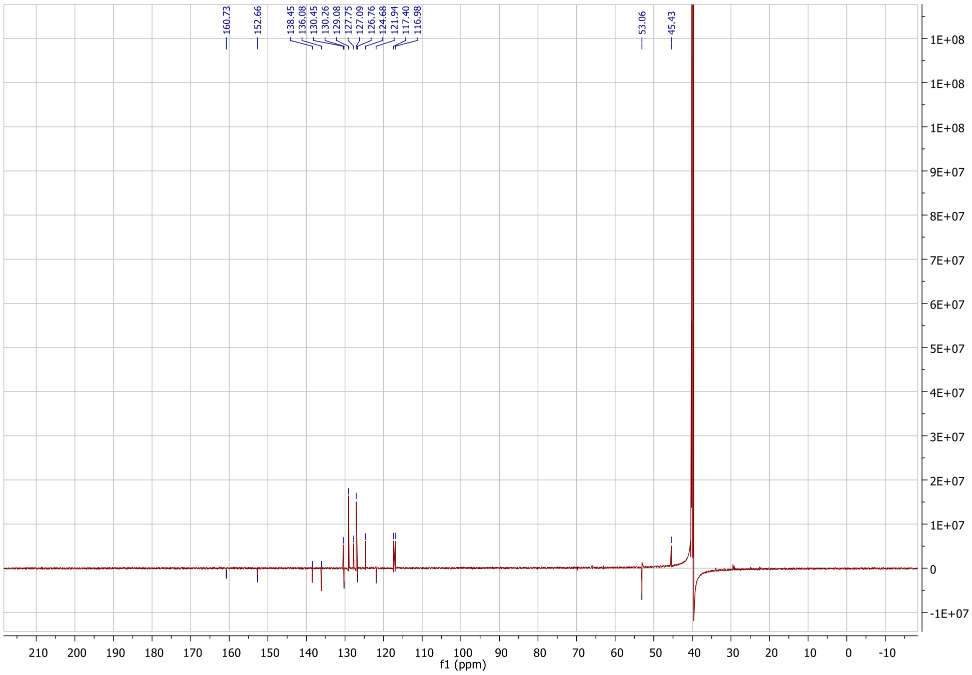
160.73
152.66
138.45
136.08
130.45
130.26
129.08
127.75
127.09
126.76
124.68
121.94
117.40
116.98
53.06
45.43
f1 (ppm)

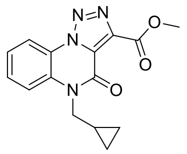

10

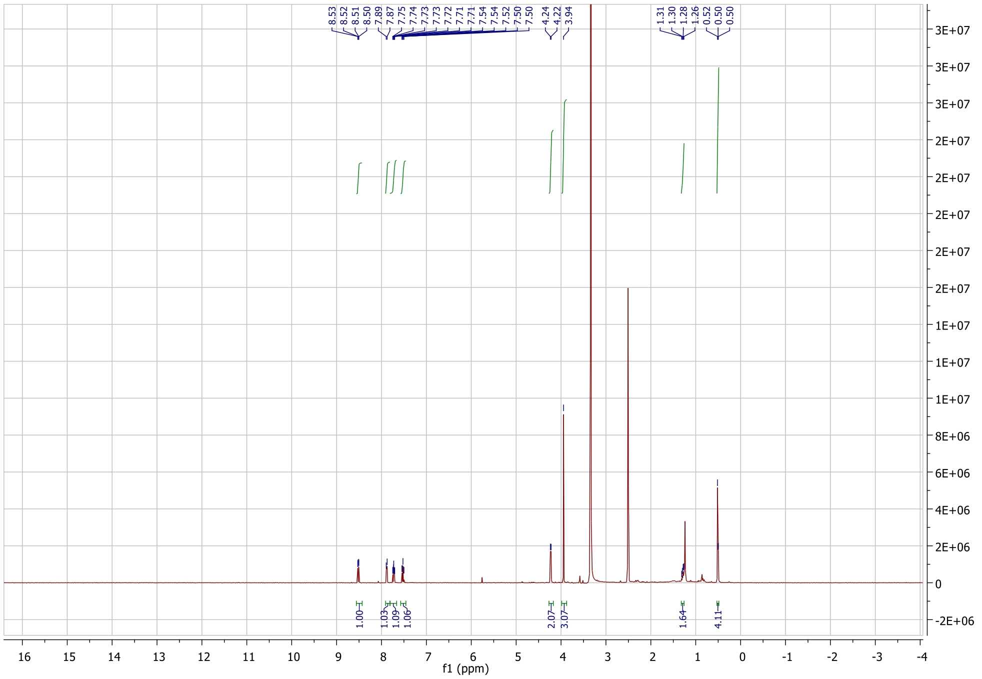
8.53 8.52 8.51 8.50 7.89 7.87 7.75 7.74 7.73 7.73 7.72 7.72 7.71 7.71 7.54 7.54 7.52 7.50 7.50 4.24 4.22 3.94
1.31 1.30 1.28 1.26 0.52 0.50 0.50
1.00 1.03 1.09 1.06 2.07 3.07 1.64 4.11
f1 (ppm)

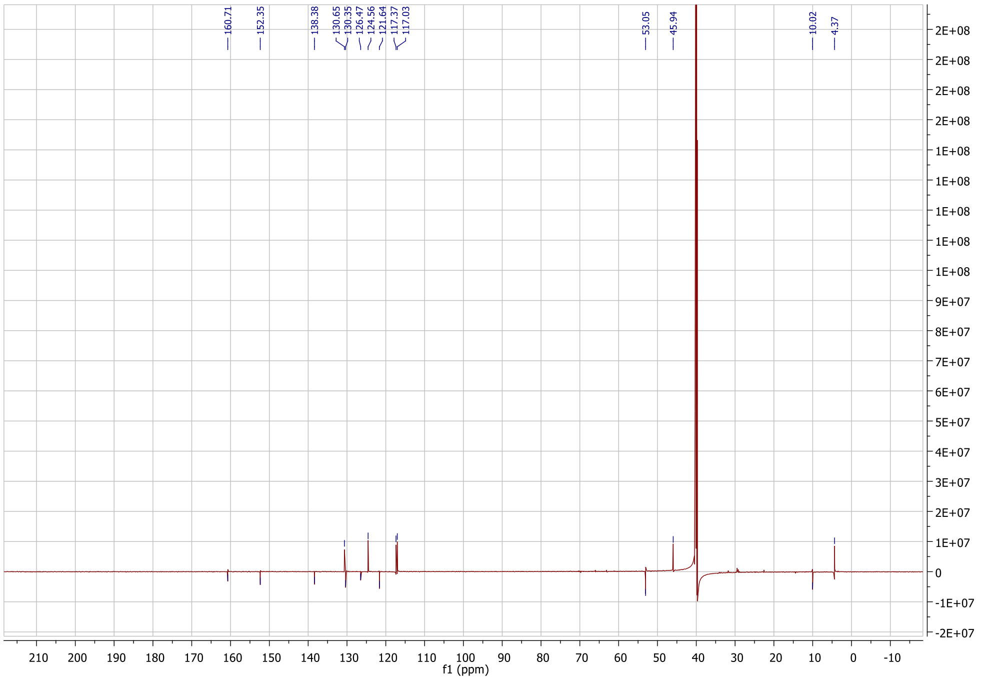
160.71 152.35 138.38 130.65 130.35 126.47 124.56 121.64 117.37 117.03 53.05 45.94 10.02 4.37
f1 (ppm)

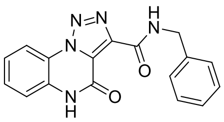

11

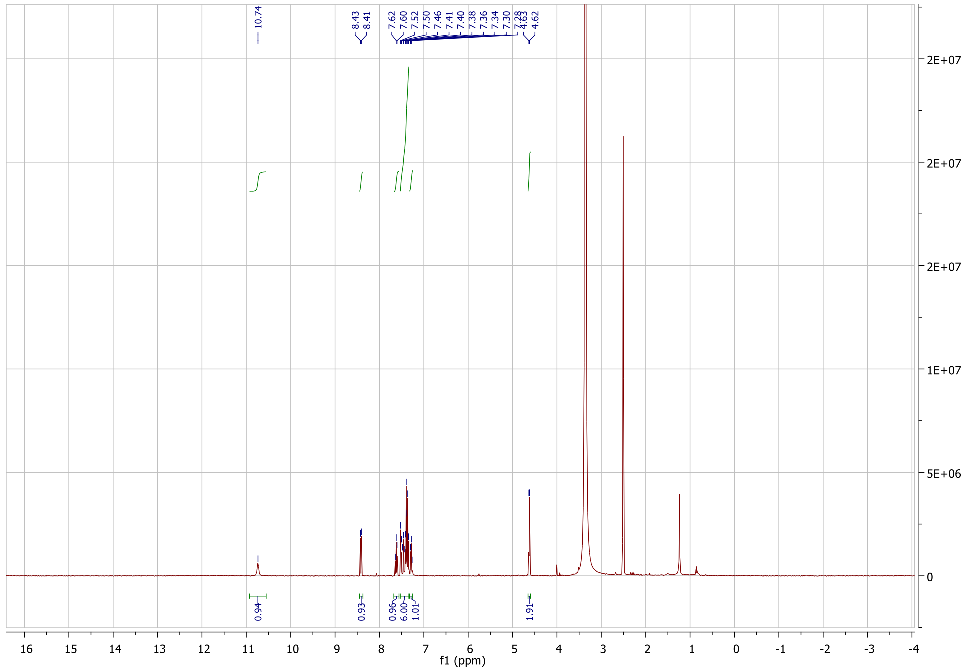

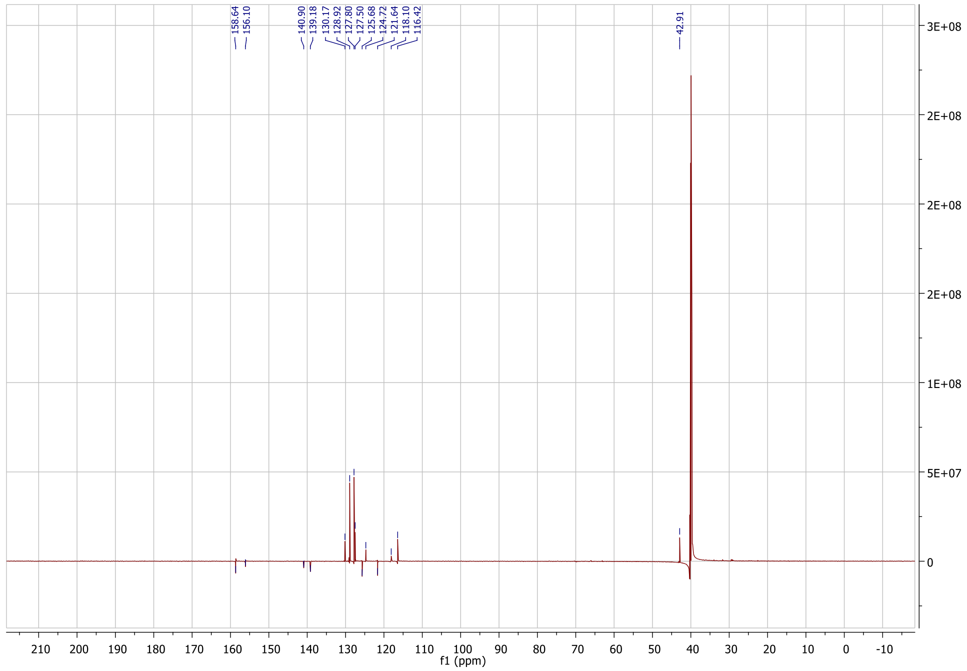

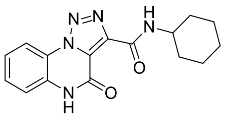
N
N
N
H
N
O
O
N
H

12

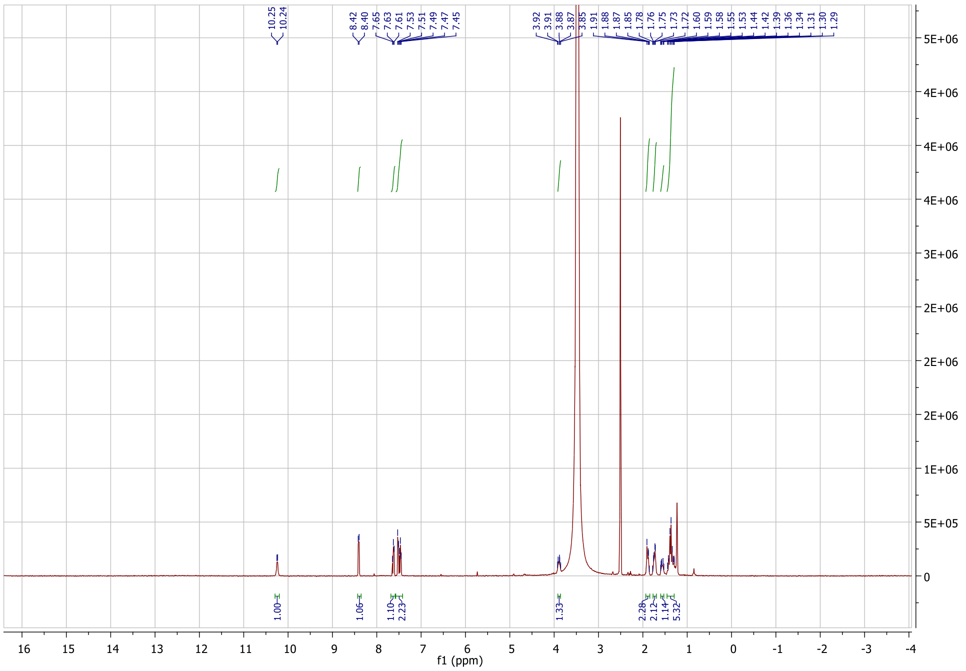
10.25
10.24
8.42
8.40
7.65
7.63
7.61
7.53
7.51
7.49
7.47
7.45
3.92
3.91
3.88
3.87
3.85
1.91
1.88
1.87
1.85
1.78
1.76
1.75
1.73
1.72
1.60
1.59
1.58
1.55
1.53
1.44
1.42
1.39
1.36
1.34
1.31
1.30
1.29
1.00
1.06
1.10
2.23
1.33
2.28
2.12
1.14
5.32
f1 (ppm)

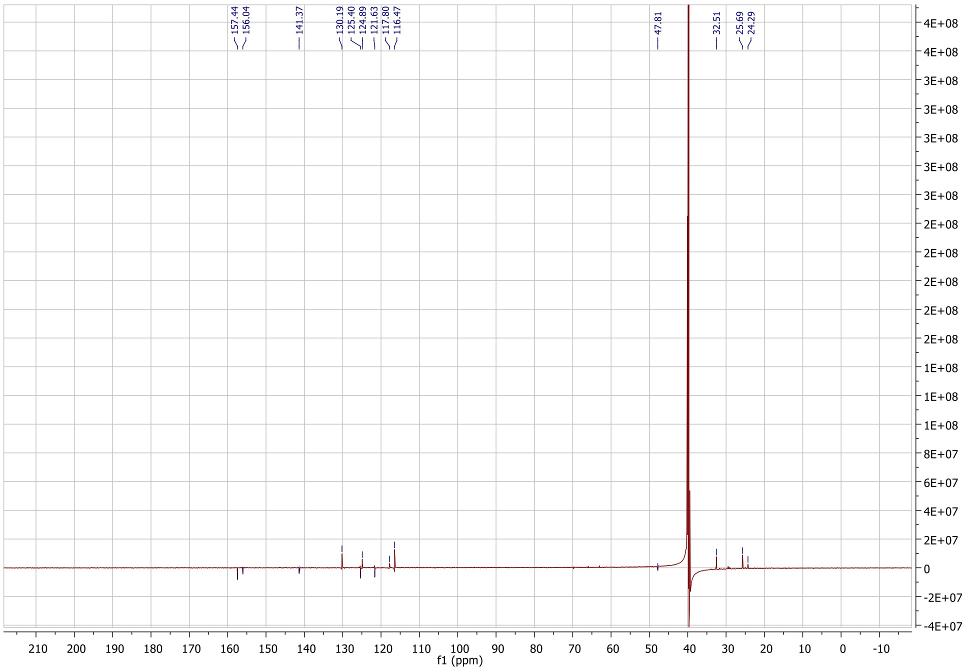
157.44
156.04
141.37
130.19
125.40
124.89
121.63
117.80
116.47
47.81
32.51
25.69
24.29
f1 (ppm)

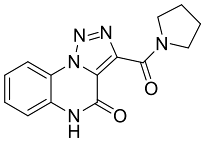
N
N
N
N
O
N
H
O

13

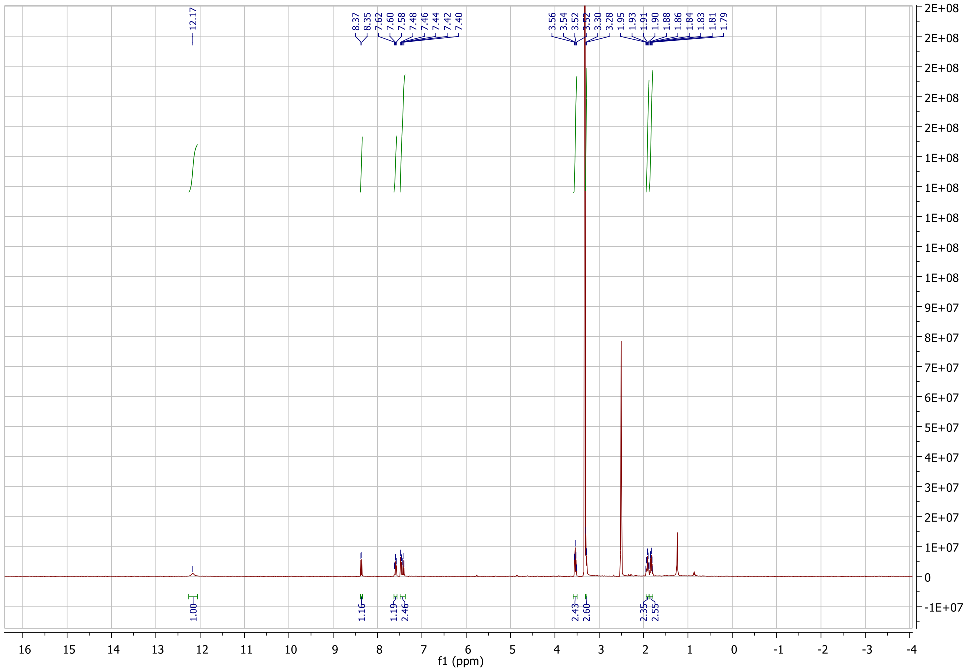
12.17
8.37
8.35
7.62
7.60
7.58
7.48
7.46
7.44
7.42
7.40
3.56
3.54
3.52
3.32
3.30
3.28
1.95
1.93
1.91
1.90
1.88
1.86
1.84
1.83
1.81
1.79
1.00
1.16
1.19
2.46
2.43
2.60
2.35
2.55
f1 (ppm)

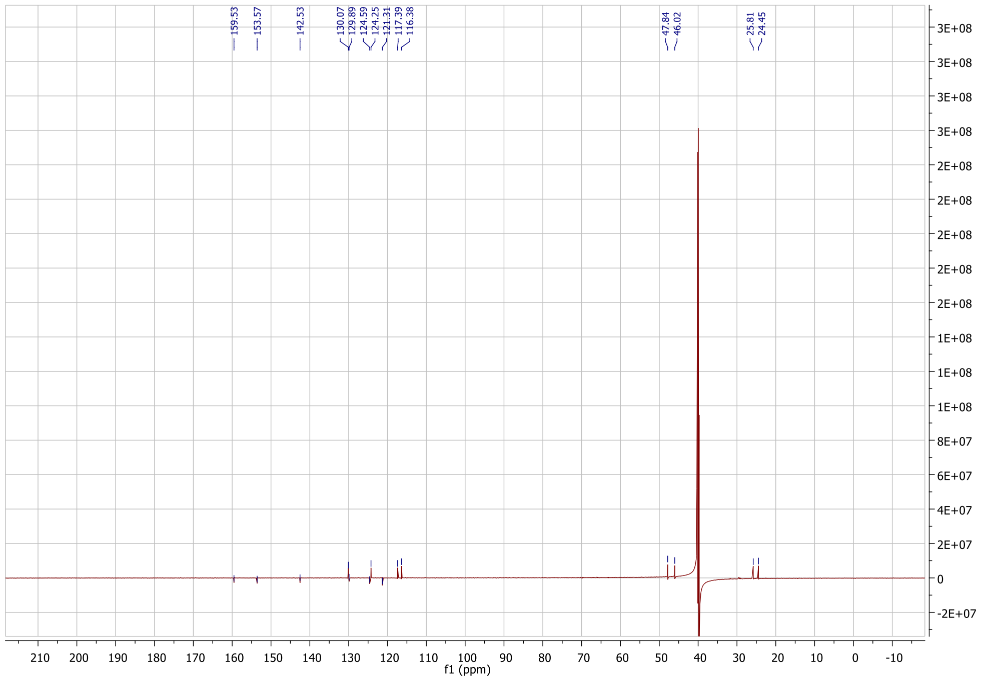
159.53
153.57
142.53
130.07
129.89
124.59
124.25
121.31
117.39
116.38
47.84
46.02
25.81
24.45
f1 (ppm)

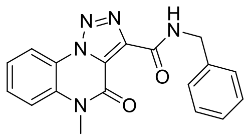

14

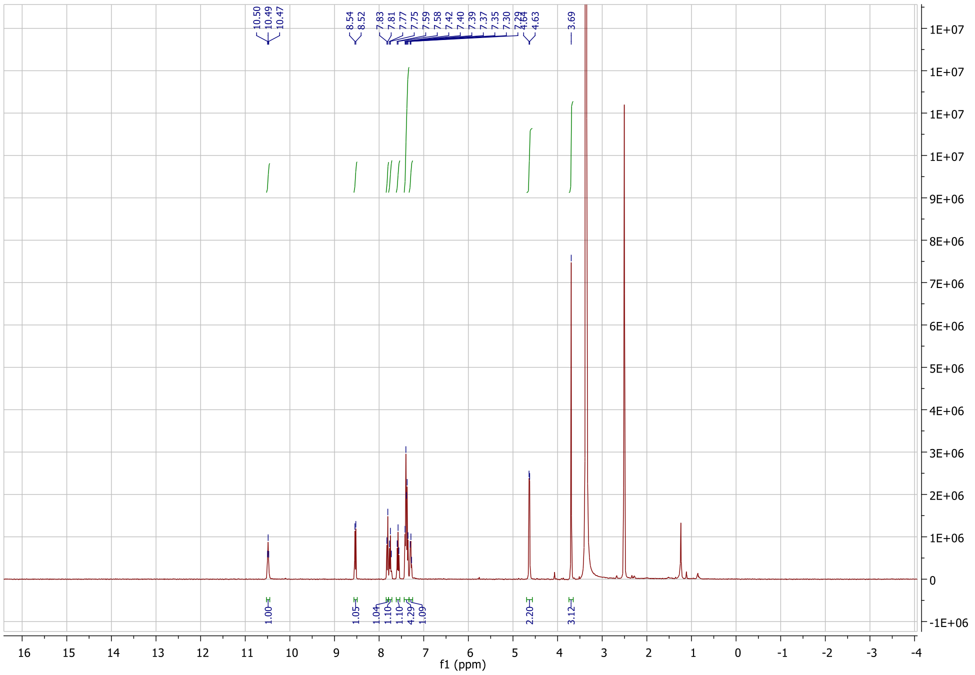
10.50
10.49
10.47
8.54
8.52
7.83
7.81
7.77
7.75
7.59
7.58
7.42
7.40
7.39
7.37
7.35
7.30
4.64
4.63
3.69
1.00
1.05
1.04
1.10
1.10
4.29
1.09
2.20
3.12
f1 (ppm)

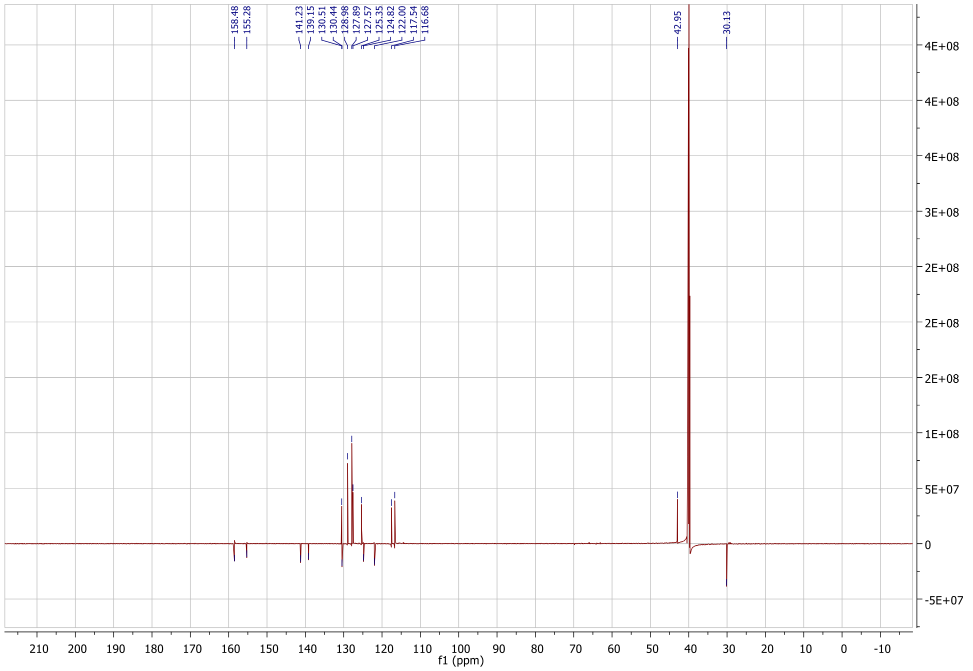
158.48
155.28
141.23
139.15
130.51
130.44
128.98
127.89
127.57
125.35
124.82
122.00
117.54
116.68
42.95
30.13
f1 (ppm)

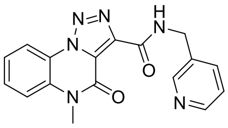

15

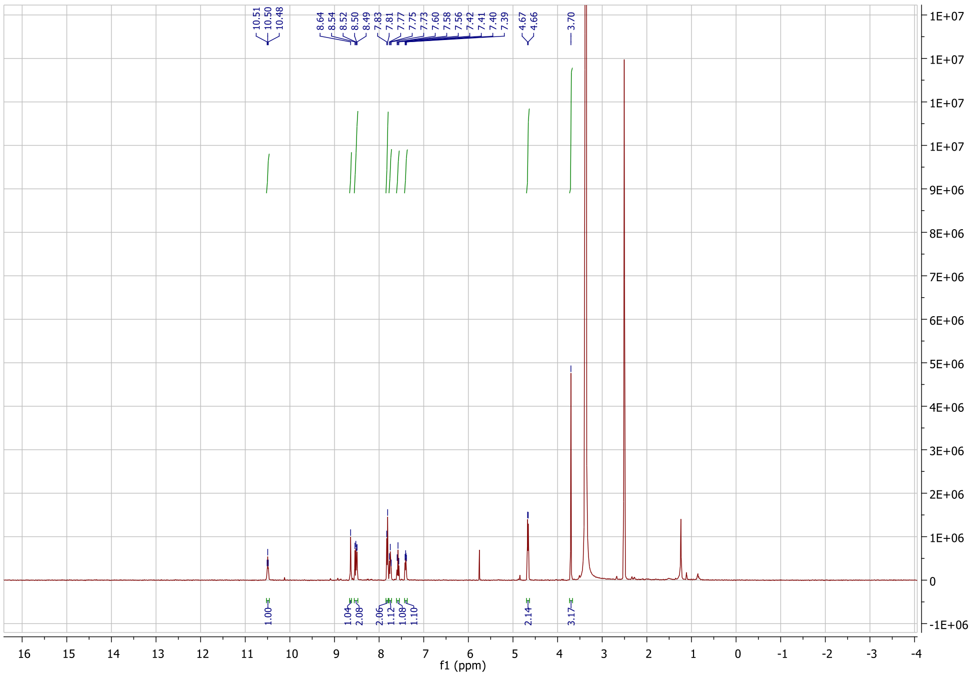
10.51
10.50
10.48
8.64
8.54
8.52
8.50
8.49
7.83
7.81
7.77
7.75
7.73
7.60
7.58
7.56
7.42
7.41
7.40
7.39
4.67
4.66
3.70
1.00
1.04
2.08
2.06
1.12
1.08
1.10
2.14
3.17
f1 (ppm)

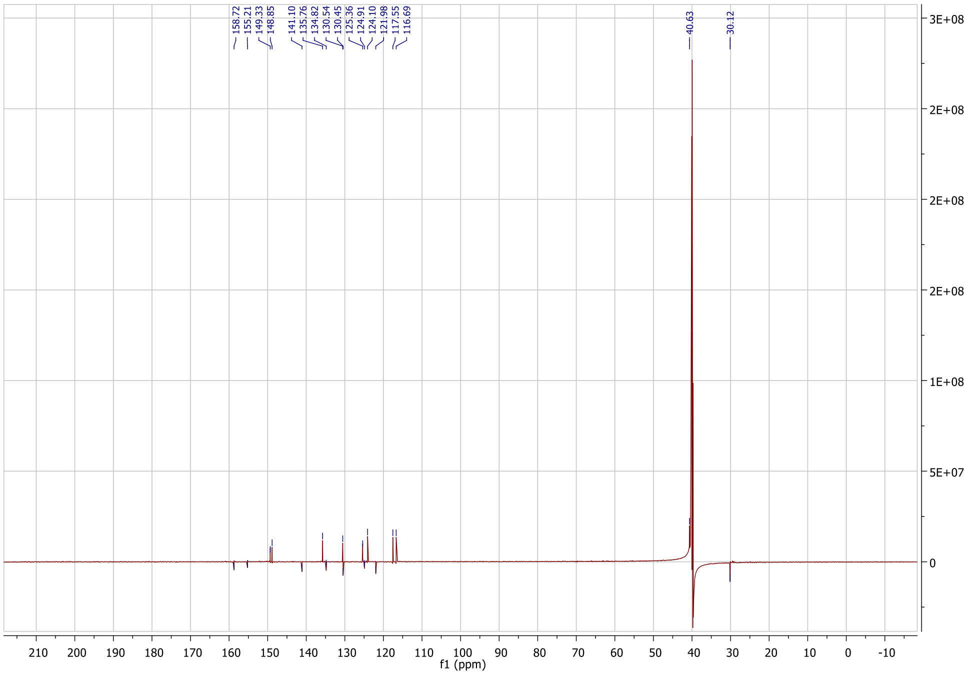
158.72
155.21
149.33
148.85
141.10
135.76
134.82
130.54
130.45
125.36
124.91
124.10
121.98
117.55
116.69
40.63
30.12
f1 (ppm)

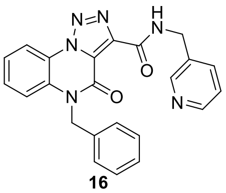

16

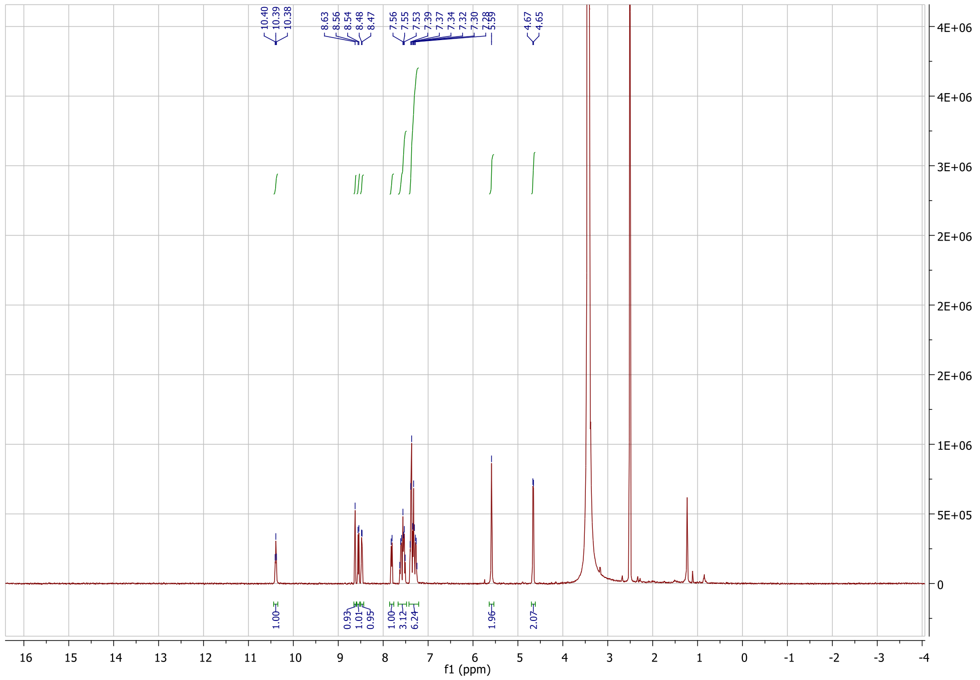
10.40
10.39
10.38
8.63
8.56
8.54
8.48
8.47
7.56
7.55
7.53
7.39
7.37
7.34
7.32
7.30
7.28
5.59
4.67
4.65
1.00
0.93
1.01
0.95
1.00
3.12
6.24
1.96
2.07
f1 (ppm)

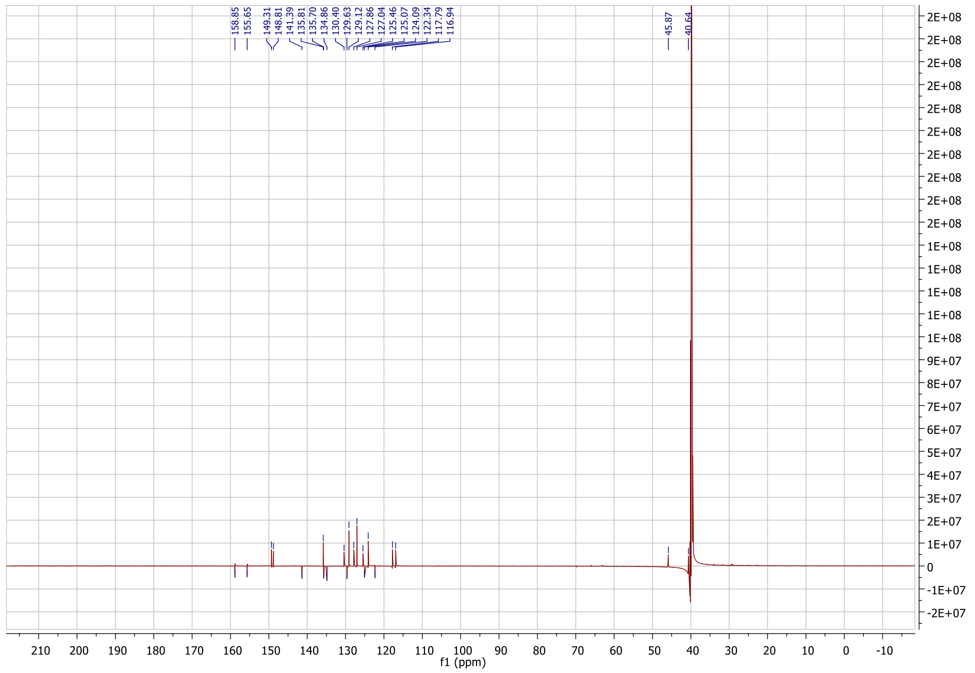
158.85
155.65
149.31
148.81
141.39
135.81
135.70
134.86
130.40
129.63
129.12
127.86
127.04
125.46
125.07
124.09
122.34
117.79
116.94
45.87
40.64
f1 (ppm)

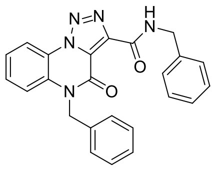
N=N
H
N
N
O
N
O

17

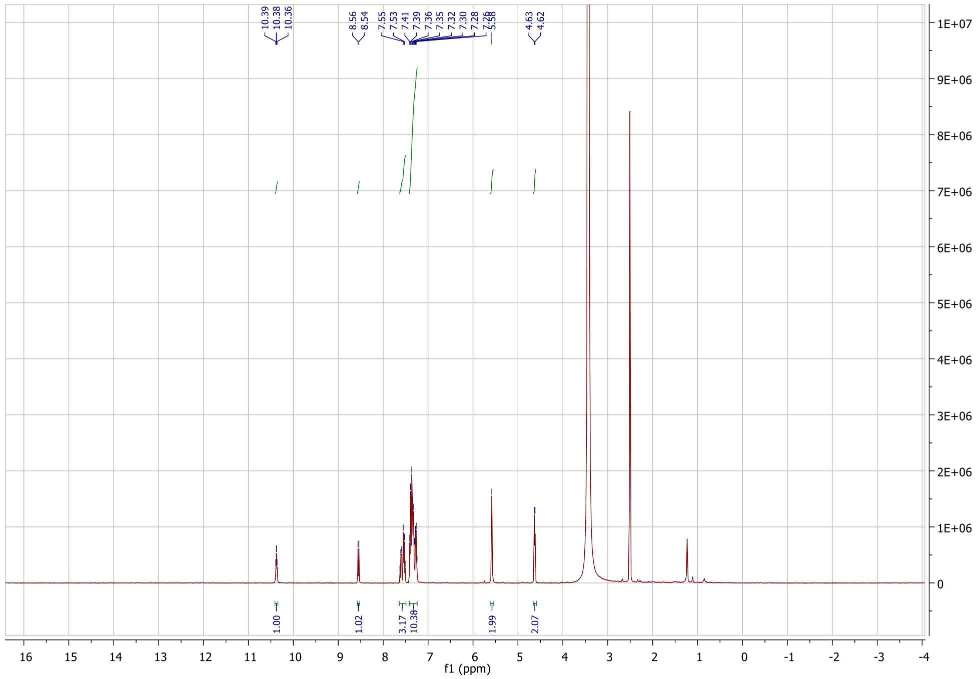
10.39
10.38
10.36
8.56
8.54
7.55
7.53
7.41
7.39
7.36
7.35
7.32
7.30
7.28
7.26
5.58
4.63
4.62
1.00
1.02
3.17
10.38
1.99
2.07
f1 (ppm)

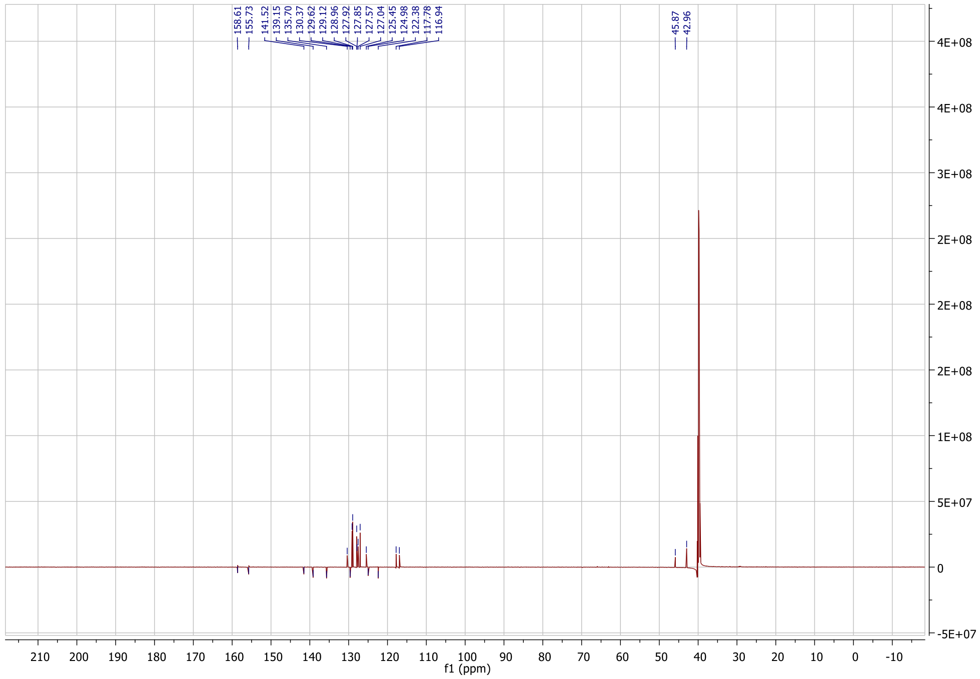
158.61
155.73
141.52
139.15
135.70
130.37
129.62
129.12
128.96
127.92
127.85
127.57
127.04
125.45
124.98
122.38
117.78
116.94
45.87
42.96
f1 (ppm)

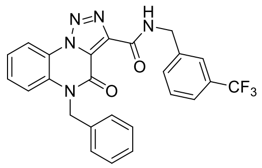

CF3

18

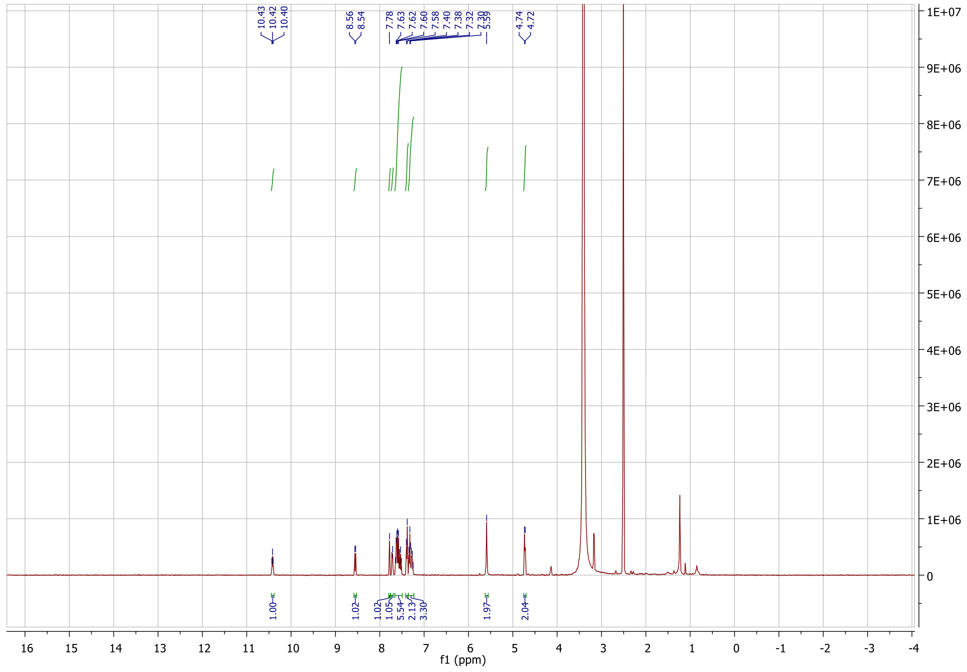

10.43
10.42
10.40
8.56
8.54
7.78
7.63
7.62
7.60
7.58
7.40
7.38
7.32
7.30
5.59
4.74
4.72
1.00
1.02
1.02
1.05
5.54
2.13
3.30
1.97
2.04
f1 (ppm)

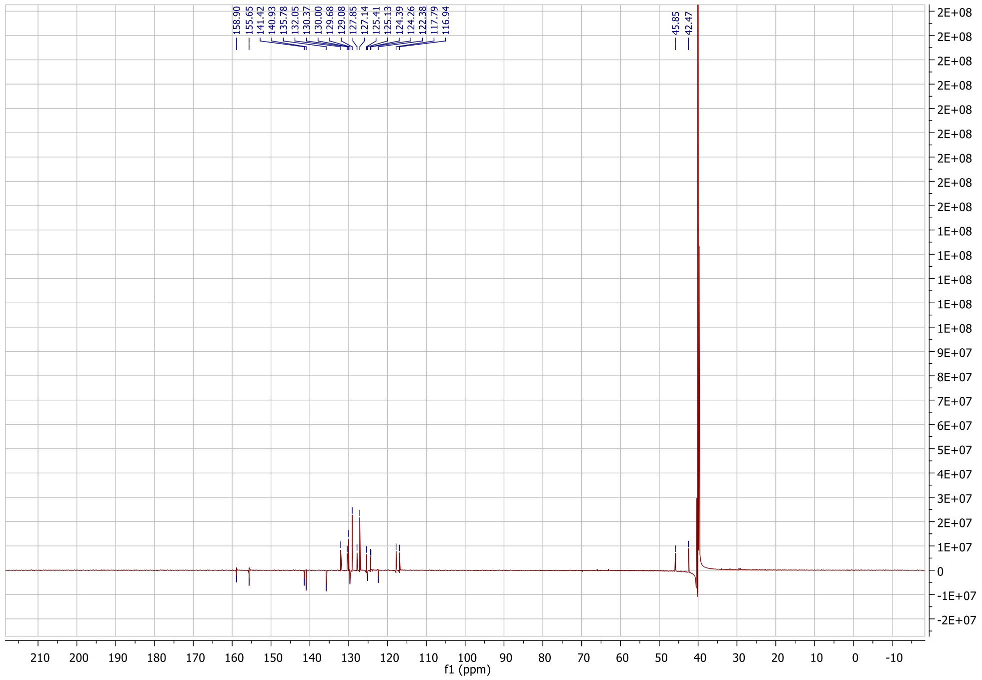

158.90
155.65
141.42
140.93
135.78
132.05
130.37
130.00
129.68
129.08
127.85
127.14
125.41
125.13
124.39
124.26
122.38
117.79
116.94
45.85
42.47
f1 (ppm)

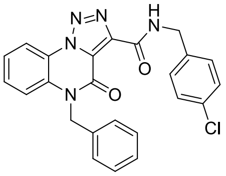
N=N
N
H
N
O
N
O
Cl

19

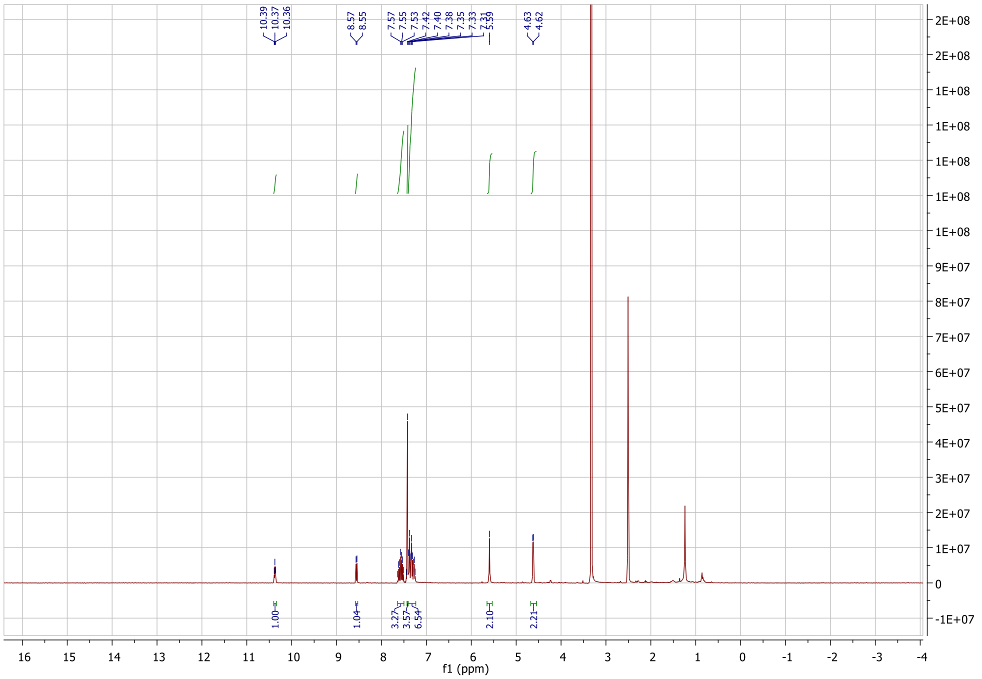
10.39
10.37
10.36
8.57
8.55
7.57
7.55
7.53
7.42
7.40
7.38
7.35
7.33
7.31
5.59
4.63
4.62
1.00
1.04
3.27
3.57
6.54
2.10
2.21
f1 (ppm)

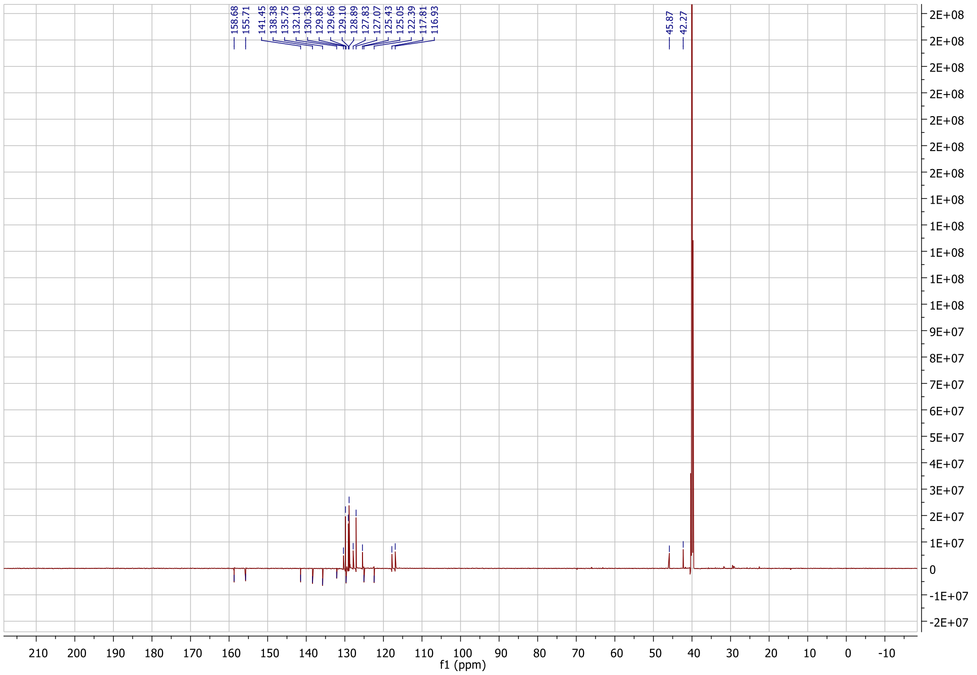
158.68
155.71
141.45
138.38
135.75
132.10
130.36
129.82
129.66
129.10
128.89
127.83
127.07
125.43
125.05
122.39
117.81
116.93
45.87
42.27
f1 (ppm)

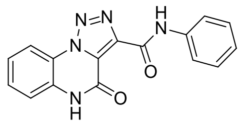

20

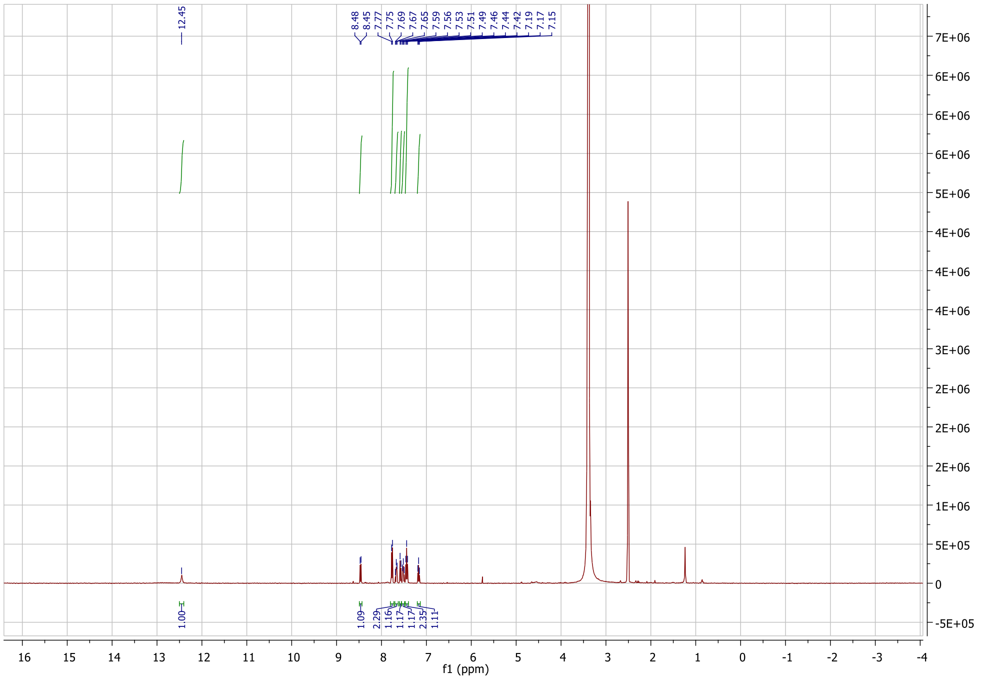
12.45
8.48
8.45
7.77
7.75
7.69
7.67
7.65
7.59
7.56
7.53
7.51
7.49
7.46
7.44
7.42
7.19
7.17
7.15
1.00
1.09
2.29
1.16
1.17
1.17
2.35
1.11
f1 (ppm)

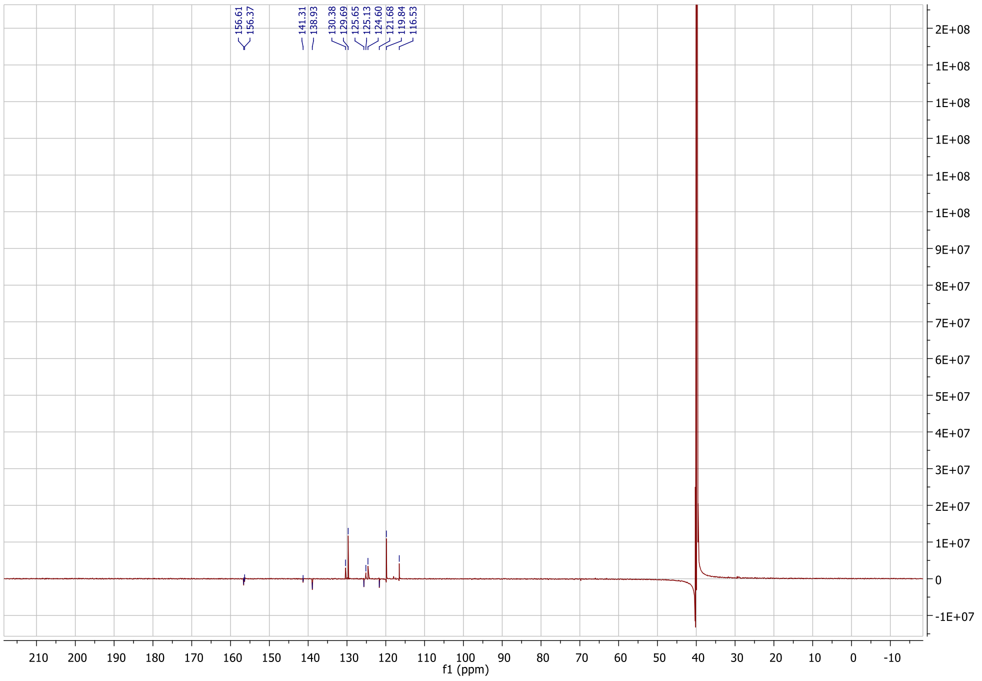
156.61
156.37
141.31
138.93
130.38
129.69
125.65
125.13
124.60
121.68
119.84
116.53
f1 (ppm)
